# Recommendations for the treatment of endometrial cancer in settings with limited resources: report from the International Gynecological Cancer Society consensus meeting

**DOI:** 10.3389/fonc.2026.1676000

**Published:** 2026-03-06

**Authors:** Fernando Cotait Maluf, Francinne T. Tostes, Henrique Alkalay Helber, Ligia A Maluf, Juliana Karassawa Helito, Renato Moretti Marques, Graziela Z. Dal Molin, Bruno Roberto Braga Azevedo, David Isla Ortiz, David Cantu, Agnaldo Lopes Silva Filho, Angélica Nogueira Rodrigues, Reitan Ribeiro, Georgia Fontes Cintra, Gustavo Guitmann, Andreia Cristina de Melo, Eduardo Paulino, Glauco Baiocchi, Leandro Santos de Araujo Resende, Michelle Samora de Almeida, Diocesio Pinto, Julio Lau de la Vega, Florencia Noll, Juliana Rodriguez, Fabio Fin, Andre Lopes, Miguel Enrique Matute Correa, Joseph S. Ng, Daniel Sanabria, Gabriel Rendon, Fernando Heredia, Ana Paula E. Galerani Lopes, Myriam Beatriz Perotta Mussi, Marcin Stanisław Bobiński, Milagros Perez Quintanilla, Jimena Meymar, David Atallah, Audrey Tsunoda

**Affiliations:** 1 Albert Einstein Israelita Hospital, São Paulo, Brazil; 2 Beneficiência Portuguesa (BP) - Mirante Hospital, São Paulo, Brazil; 3 Department of Surgical Oncology, Oncoclínicas, Curitiba, Brazil; 4 Department of Oncological Gynecology, National Institute of Cancerology, Mexico City, Mexico; 5 Federal University of Minas Gerais, Belo Horizonte, Minas Gerais, Brazil; 6 Department of Surgical Oncology, Erasto Gaertner Hospital, Curitiba, Brazil; 7 Georgia Fontes Cintra, Instituto Brasileiro de Controle do Cancer, São Paulo, Brazil; 8 Brazilian National Cancer Institute, Rio de Janeiro, Brazil; 9 Gynecologic Oncology, Antonio Cândido de Camargo Cancer Center, São Paulo, SP, Brazil; 10 Department of Surgical Oncology, Oncoclínicas, Brasilia, DF, Brazil; 11 Federal University of Sao Paulo, Sao Paulo, Brazil; 12 InORP Group Oncoclínicas, Ribeirão Preto, Brazil; 13 GYNECARE Women's Health Clinic, Guatemala City, Guatemala; 14 Sanatorio Allende Cerro, Cordoba City, Argentina; 15 National Cancer Institute (INC), Bogotá, Colombia; 16 Centro de Oncologia do Paraná, Paraná, Brazil; 17 Onco Prevee, Lima, Peru; 18 Division of Gynecologic Oncology, National University Cancer Institute, Singapore, Singapore; 19 Hospital de San Jose Fundación Universitaria de Ciencias de la Salud Bogotá, Bogotá, Colombia; 20 Gynecologic Oncology, Instituto de Cancerología, Bogotá, Colombia; 21 Oncología Ginecológica en el Instituto Clínico de la Fundación Arturo López Pérez, Providência, Chile; 22 Sultan Qaboos Cancer Care and Comprehensive Centre em Mascate, Seeb, Oman; 23 Hospital Italiano de Buenos Aires, Buenos Aires, Argentina; 24 Independent Laboratory of Translational Medicine, Department of Clinical Genetics, Medical University of Lublin, Lublin, Poland; 25 Department of Gynecology, Obstetrics and Perinatology, Pope John Paul II Independent Public Provincial Hospital in Zamość, Zamość, Poland; 26 M2 Innovations Ltd., Zamość, Poland; 27 Gynecologic Oncology and Breast Cancer at Medical Center ABC, Mexico City, Mexico; 28 Kenes Group, Geneva, Switzerland; 29 Hôtel Dieu de France University Hospital, Women's Cancer Institute, Saint Joseph University, Beirut, Lebanon; 30 HCor, Ecomedical Center, Hospital Erasto Gaertner, PUCPR, Curitiba, Brazil

**Keywords:** consensus, endometrial cancer, guideline, gynecological cancer, gynecological malignancies, low resources countries, resource limitation, resource-stratified guidelines

## Abstract

**Introduction:**

Given the high disparities present in cancer care worldwide and even more challenging infrastructure and access for low- and middle-income countries, adhering precisely to international guidelines has become a challenging and complex task. Recommendations from an independent multidisciplinary panel of experts from 13 countries, including medical oncologists, pathologists, surgeons, and radiation oncologists, who met during IGCS to address some of these challenges.

**Methods:**

The panel met in New York City in September of 2022 during the International Gynecological Cancer Society Congress and was composed of specialists from developing countries in Africa, Asia, Eastern Europe, Latin America, and the Middle East. The panel addressed 103 questions and provided recommendations for the management of early, locally advanced, recurrent, and/or metastatic endometrial cancer. The questions were carefully developed by the group and specifically directed to, and answered by, specialists according to their respective areas of expertise. Consensus was defined as at least 75% of the voting members selecting a particular recommendation, whereas a majority vote was considered when one option garnered between 50.0% and 74.9% of votes. Resource limitation was defined as any issues limiting access to qualified surgeons, contemporary imaging or radiation-oncology techniques, antineoplastic drugs, or funding for providing modern medical care.

**Results:**

Eighteen of the 109 (16.5%) questions presented to the panel reached consensus, whereas a majority vote was reached for 43 (39.4%) additional questions. The recommendations for the remaining questions were considerably heterogeneous and were considered experts opinion only.

**Conclusion:**

Establishing guidelines with recommendations in areas with resource limitations may help healthcare providers and improve patient care around the world.

## Introduction

Endometrial cancer still accounts for a significant public health problem, with an increasing incidence in low- and middle-income countries and ranking as the leading cause of gynecologic cancer mortality in high-income countries. A total of 420,242 new cases were diagnosed in 2022, with the incidence of cases under the age of 40 doubling its frequency. Most concerning over the past decade the overall incidence has increased by 132%. The rising incidence is attributed to the increase of its two most common risk factors: obesity and population aging (1, 2).

The treatment landscape of endometrial cancer has substantially improved, since the addition of immunotherapy to the backbone chemotherapy regimen in advanced disease, the introduction of anti-HER2 therapies in some specific tumor types that express HER2, and the improvement in radiation therapy and surgical techniques. In low to middle-income countries, the same benefit may be challenging to achieve due to limited access to oncology medications and surgical and radiation procedures. Cancer survival rates tend to have high disparities due to multifactorial causes such as region, race, socioeconomic, biological, and cultural factors (3). As shown in research performed in low-income areas in the United States, patients treated in high-income areas have improved healthcare access to established treatments, research trials, funding and regular follow-up during therapy to manage possible toxicities (4). To date, guidelines from different international gynecological societies generally do not cover the diagnosis and management of endometrial cancer in areas with limited resources, causing uncertainty regarding adequate management of actions (5). Therefore, to reduce heterogeneity in the quality of cancer care in low-income countries, we proposed developing adaptive guidelines through consensus panels during high-level international meetings. This article is part of a series of reports from that consensus meeting, convened under the auspices of the International Gynecological Cancer Society.

## Methods

### Composition, organization, and objectives of the panel

The questions addressed by the panel were proposed by a 15-member committee as the most relevant for decision-making in areas facing resource limitations. The panel, composed of invited specialists in gynecological oncology from 10 developing countries in Africa, Asia, Eastern Europe, Latin America, and the Middle East, aimed to provide recommendations on salient issues that affect the management of endometrial cancer in these areas (**Supplementary Table 1**). Certain regions and countries were underrepresented or not represented, which may limit the generalizability of the recommendations to all low−resource settings. Panel members were opinion leaders in pathology, gynecology, oncological surgery, medical oncology, radiation oncology, and radiology in their respective countries. Using an electronic voting system, the panel answered the questions in polling sessions held on September 30th, 2022 during the International Gynecological Cancer Society Congress in New York, United States of America.

The 15−member steering committee generated and prioritized the clinical questions by synthesizing recommendations and key decision points from established, high−impact gynecologic−oncology guidelines and staging systems. Primary sources used to frame and harmonize the questions included the NCCN Clinical Practice Guidelines in Oncology, the ESMO and ESGO/ESTRO/ESP guideline documents.

One polling session with multiple-choice questions was scheduled for each main topic that constitutes the subheadings described below, with specialists in each area responding to questions corresponding to their respective areas of expertise. When answering each multiple- choice question, panel members were instructed to consider that their recommended intervention was approved and available, with no contraindications in the scenario described by the corresponding question. Moreover, recommendations were to be given for non-frail patients (defined as having an Eastern Cooperative Oncology Group [ECOG] performance status between 0 and 2) and for patients with endometrial cancer. Finally, the staging classification used throughout was the latest one provided by the International Federation of Obstetrics and Gynecology.

### Definition of resource limitation

The World Bank classifies country economies into four groups according to their average income: high, upper-middle, lower-middle, and low (16). Even though the panel included members from countries that may belong to different income groups, the socioeconomic framework used herein relates to the availability of ideal resources. This is especially relevant in some of the countries represented, which have heterogeneous healthcare systems. Regardless of the situation in individual countries, the current work focuses on “area” rather than “country”, under the assumption that medical practice may not necessarily be constrained in a whole country and still be subject to resource limitation in some of its areas or settings. Finally, resource limitation was broadly defined as limited access to qualified surgeons, contemporary imaging or radiation- oncology techniques, antineoplastic drugs, or overall funding for providing state-of-the- art health care.

### Responsibilities

These guidelines represent a statement of evidence and consensus from the authors regarding their perspectives on currently accepted approaches to the management of patients with endometrial carcinoma, particularly in low- and middle-income countries where resources and access to therapies may be severely limited. Clinicians applying or consulting these guidelines are expected to exercise independent medical judgment within the context of individual patient circumstances when determining care or treatment. The guidelines make no warranties, express or implied, regarding their content, use, or application, and the authors disclaim any responsibility for the consequences of their use in clinical practice.

### Statistical analysis

Results for each of the questions addressed by the panel are presented descriptively and grouped according to clinical setting or issue. If at least 75% of the voting members selected a particular option for a given question, consensus was present. If between 50% and 74.9% of the voting members selected a specific choice, this was considered a majority vote, but no consensus. The percentages shown herein do not consider the response option “unqualified to answer” in their denominator, which was available at the meeting. On the other hand, the response option “abstain” was considered in the denominator for each question; this option referred to cases for which a member felt impeded from providing a qualified response for reasons other than lack of knowledge, including the presence of conflicts of interest.

## Results

### Section 1: initial assessment

Six questions addressed initial assessment in resource-limited settings. Consensus was achieved for two (**Supplementary Table 2**). The most frequently endorsed imaging modalities were pelvic and abdominal computed tomography (CT) and chest X-ray, settings without CT access, pelvic and abdominal ultrasound with chest X-ray, was preferred.

For early stage endometrioid subtype grades 1 and 2, no consensus was achieved. Still, the most suggested recommendation were (1) a physical exam (PE), pelvic and abdominal ultrasound, and chest X-ray (35%); or (2) PE, pelvic/abdominal CT when available(35%). When CT scan was unavailable, the majority (53%) recommended PE, pelvic/abdominal ultrasound, and chest X-ray, whereas 37% supported omitting chest imaging.

For early-stage high-grade histologies, a clear majority 74% favored pelvic/abdominal CT and chest X-ray.

**Figure 1:**
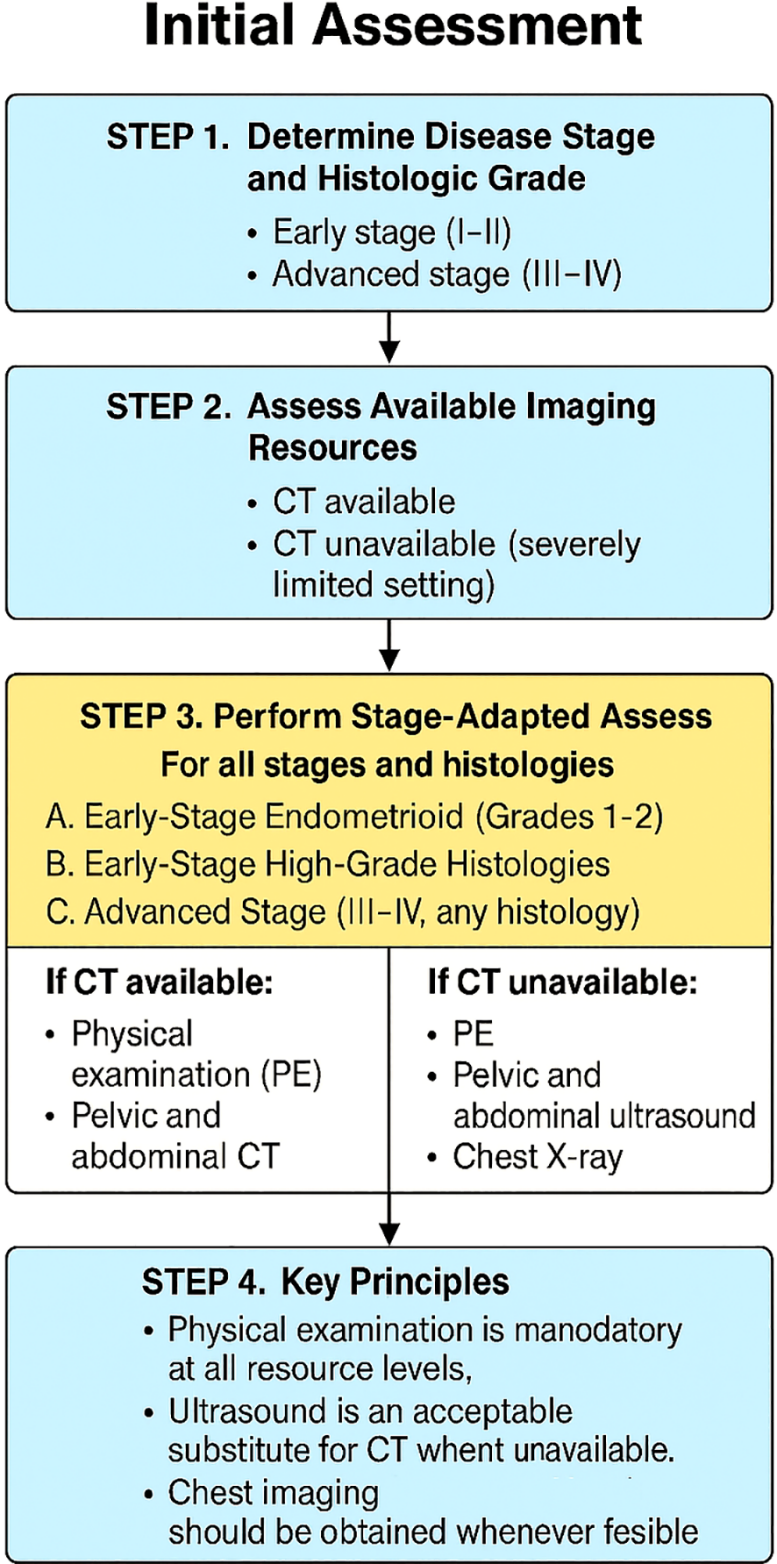
Stage-adapted framework for initial assessment of endometrial cancer in resource-limited settings. The algorithm summarizes recommendations according to disease stage, histology, and availability of CT imaging. PE, physical examination; CT, computed tomography.

For stages III-IV, 90% recommended physical exam, pelvic/abdominal CT, and chest X-ray. Formal consensus was also reached that pelvic and abdominal ultrasound is an acceptable alternative to CT when unavailable. **Figure 1** provides a concise summary synthesizing the consensus recommendations presented in this article.

Recommendation by consensus vote:

- For patients with stage III-IV endometrial cancer, initial assessment in an area of severely limited resources must include PE, pelvic and abdominal CT, and chest X-ray.
- For patients with stage III-IV endometrial cancer, initial assessment in an area of severely limited resources and no access to CT, patients should undergo PE, pelvic and abdominal ultrasound, and chest X-ray.

Recommendation by majority vote:

- For patients with early-stage endometrioid subtype grades 1 and 2 endometrial cancer and early-stage high-grade histologies, initial assessment in an area of severely limited resources should include PE, pelvic and abdominal CT and chest X-ray.
- Likewise, for patients in areas with severely limited resources and no access to CT, the minimum evaluation includes PE, pelvic and abdominal ultrasound, and chest X-ray.

### Section 2: surveillance of endometrial cancer

Nine questions were asked about surveillance of endometrial carcinoma. Consensus was achieved in only one of those questions (**Supplementary Table 3**).

For questions related to surveillance, two achieved consensus and one had a clear majority vote. Both supported the same recommendation: for stages I-IV after curative treatment, as well as for recurrent endometrial cancer after response to salvage local therapy the follow-up should be every 3 months in the first 2 years, after that, every 6 months until 5 years from treatment. 

Regarding surveillance for stages I-II, no formal consensus was achieved. As 40% of panelists supported clinical examination only as the surveillance method.

**Figure 2:**
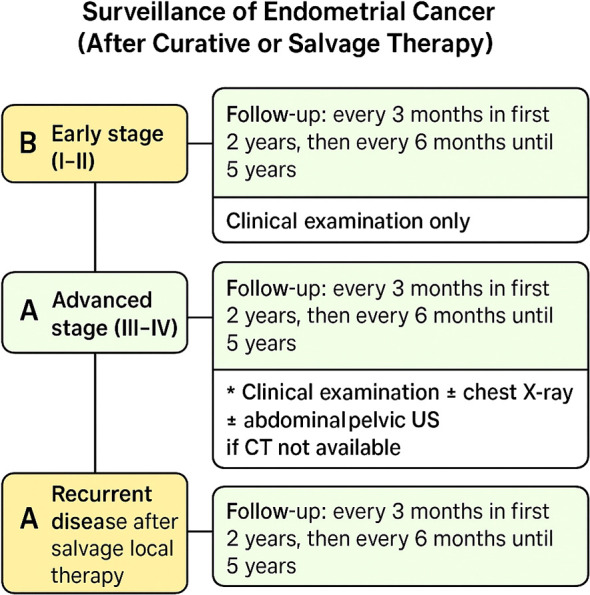
Post-treatment surveillance algorithm for endometrial cancer according to stage and recurrence status. Follow-up intervals and recommended evaluations are summarized for early-stage, advanced-stage, and recurrent disease. CT, computed tomography; US, ultrasound.

Stages III-IV treated with curative treatment and recurrent endometrial cancer treated with salvage local therapy had considerable heterogeneity in the responses with a preponderance for using chest X-ray, clinical examination, and abdominal and pelvic US when CT scan not available. **Figure 2** provides a concise summary synthesizing the consensus recommendations presented in this article.

Recommendation by consensus vote:

- For stages III-IV, after curative treatment and recurrent endometrial cancer after response to salvage local therapy optimal follow-up should be done every 3 months in the first 2 years, and afterwards every 6 months until 5 years from treatment.

Recommendation by majority vote:

- For early stage I-II undergoing curative treatment, the minimum acceptable frequency of follow-up is every 3 months in the first 2 years, and after that, every 6 months until 5 years from treatment.

### Section 3: initial surgical approach

Sixteen questions were asked about initial surgical assessment for endometrial cancer, and formal consensus was present in four of those questions (**Supplementary Table 4**). Under resource limited-conditions, 77% of panelists considered intraoperative gross examination useful. In patients with endometrioid grade 1 and 2, ECOG 2 or more and/or severe comorbidities in an area with untrained oncology surgeons, a clear majority (81%) endorsed total abdominal hysterectomy (TAH) and bilateral salpingoophorectomy (BSO). The same approach was chosen by 82% of the voters when radiation therapy was not available, and by 86% when both radiation and specialized surgeons were absent.

**Figure 3:**
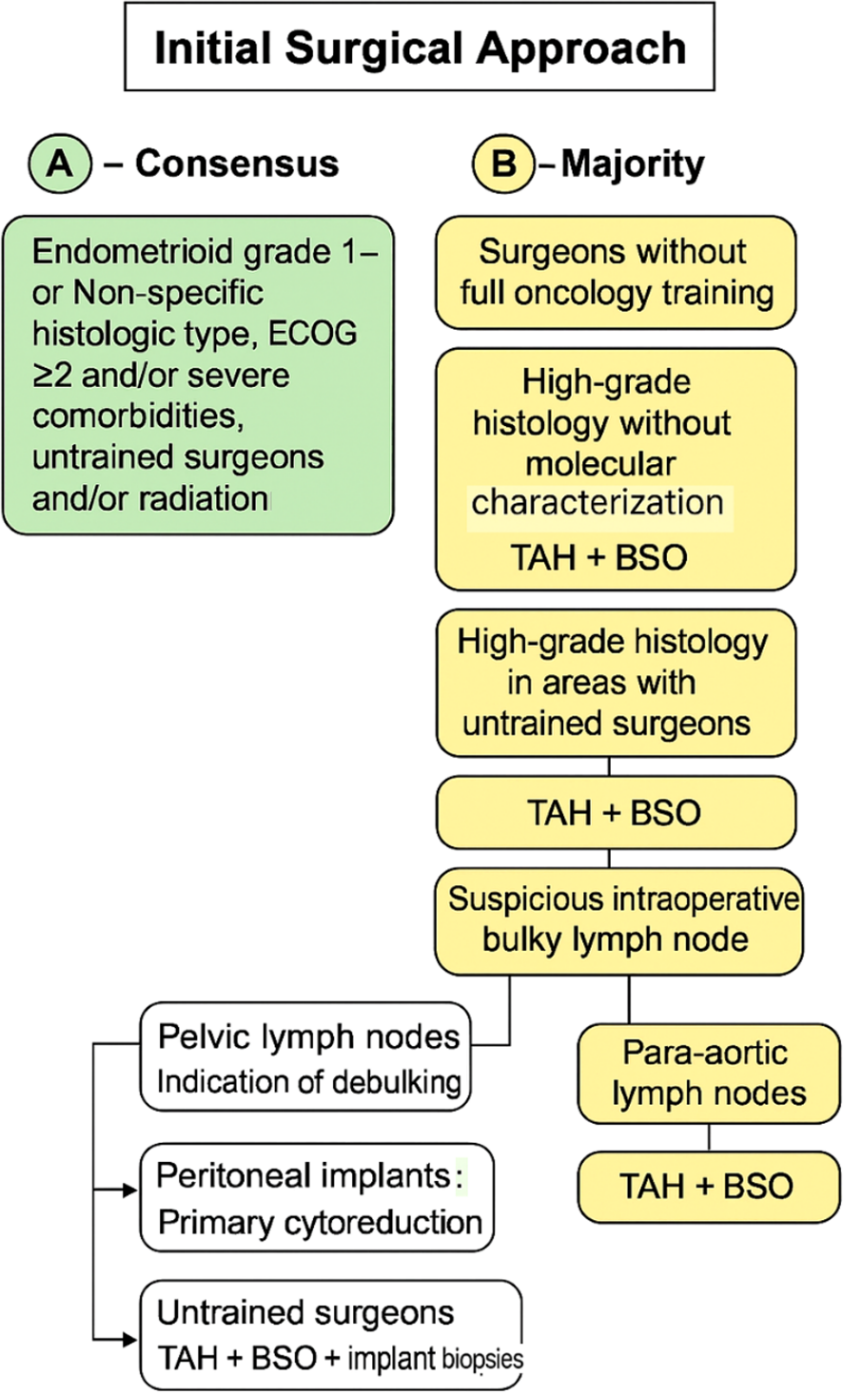
Initial surgical approach for endometrial cancer based on consensus and majority recommendations. The algorithm summarizes management according to histologic grade, surgeon expertise, and intraoperative findings, including indications for TAH + BSO and lymph node assessment or cytoreduction. TAH, total abdominal hysterectomy; BSO, bilateral salpingo-oophorectomy; ECOG, Eastern Cooperative Oncology Group performance status.

A majority vote was present for six out of the 16 surgical questions. The most frequent type of incision recommended with untrained oncology surgeons was a longitudinal laparotomy for 50% of panelists. In patients with non-specified histologic/molecular type or grade, 50% recommended TAH and BSO, regardless of myometrial invasion. Similarly, for endometrioid grades 1–2 the same surgical approach was designated by 57% of panelists. Notwithstanding, for high-grade histologies, 55% of panelists preferred performing TAH + BSO and systematic pelvic and para-aortic lymph node dissection as a preferred surgical approach. Whereas for areas with a lack of specialty surgical training, 57% opted for a less aggressive surgery not entailing lymph node dissection. For patients who presented with severe comorbidities and decreased performance status, 74% voted in favor of TAH and BSO as the best approach. Concerning the intra-operative approach for suspicious bulky lymph nodes, when surgeons did not have full surgical training, a majority (50%) voted for debulking if the only suspicion was pelvic involvement. On the subject of peritoneal implants, the majority of panelists opted for primary cytoreduction if surgically feasible, whereas in areas with no specialized training, the best approach remains TAH, BSO, and random biopsies. **Figure 3** provides a concise summary synthesizing the consensus recommendations presented in this article.

Recommendation by consensus vote:

- For endometrioid uterine cancer, grades 1 and 2, ECOG 2 or more, and/or severe comorbidities in an area with untrained oncology surgeons and/or radiation therapy the best single approach should be TAH and BSO.
- In an area of severely limited resources, intraoperative gross examination should be offered.

Recommendation by majority vote:

- In areas where surgeons do not have full surgical oncology training, longitudinal laparotomy should be the best surgical approach for endometrial cancer.
- For non-specific histologic type, grade, or molecular subtype and regardless of myometrial invasion in undertrained surgical areas TAH + BSO should be offered, the same applied for endometrioid grades 1 and 2 and non-specified myometrial invasion.
- For high-grade histologies with no molecular characterization TAH, BSO, and systematic pelvic and para-aortic lymph node dissection should be the first option unless there is no specialized surgical oncology training, then systematic lymph node dissection should be omitted.
- In the case of intra-operative suspicious bulky pelvic lymph nodes, there is an indication of debulking.
- If there is suspicion of bulky involvement of para-aortic lymph nodes, no lymph node assessment was recommended.
- For peritoneal implants, primary cytoreduction if surgically feasible is advised, however in situations where there are no surgeons with full training in oncology, the acceptable approach is to perform TAH, BSO and implant biopsies.

### Section 4: adjuvant treatment for endometrial cancer

Seventeen questions were asked about adjuvant treatment, but consensus was present for only one question (**Supplementary Table 5**).

In surgically staged low-risk endometrial cancer in areas of severe resource limitation and no molecular characterization, the minimum acceptable adjuvant strategy is observation.

The adjuvant treatment of intermediate-risk endometrial cancer, neither formal consensus nor a majority vote was achieved. A total of 33% of panelists selected brachytherapy alone as the best option, 19% external radiation therapy alone, and 19% external radiation and brachytherapy. Similarly, the panel remained divided among these two options when no lymph node staging was performed, whereas, when radiation was unavailable in the same scenario, 35% voted for observation only and 31% for chemotherapy alone. When asked about the minimum acceptable adjuvant external beam radiation technique in resected early-stage endometrial carcinoma, 36% voted for conventional radiation, and 23% for external beam radiation with cobalt machines.

Moreover, a clear majority of votes was achieved for six questions as follows:

When asked about adjuvant brachytherapy techniques for intermediate-risk endometrial carcinoma the majority of panelists choose two-dimensional radiothepray (2D). As for adjuvant chemotherapy for high-grade histologies, 60% voted in favor of the treatment. When radiation therapy was not available, the percentage went up to 71% in favor of chemotherapy alone. In institutions where only conventional radiation therapy is available, the majority of panelists (56%) agree with primary external conformal radiotherapy for the early stages. A total of 63% of panelists also agree that, in that same scenario, adjuvant external conformal radiotherapy could be offered in the adjuvant setting of early stages.

When asked about adjuvant or primary external radiotherapy with cobalt machine for early-stage endometrial cancer, the panelists did not reach a consensus or a majority. On the subject of chemotherapy for stage I-II high-risk endometrial cancer with no cisplatin contra-indication, 29% voted in favor of carboplatin and paclitaxel every 3 weeks, and 29% voted for cisplatin and paclitaxel every 3 months.

**Figure 4:**
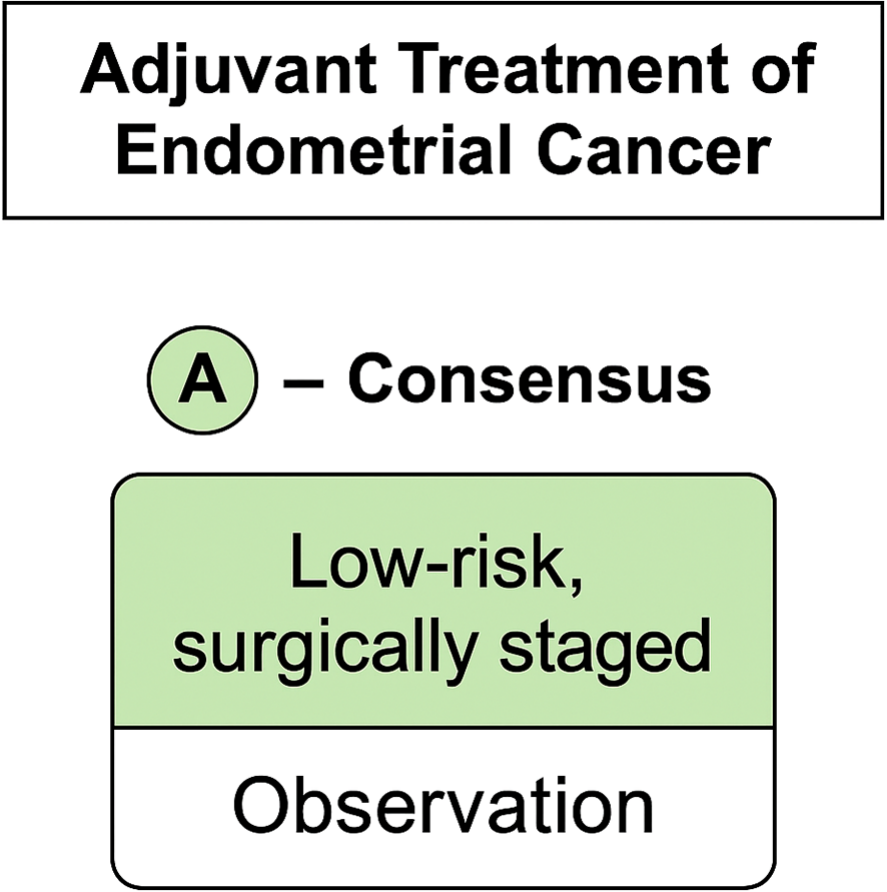
Adjuvant treatment recommendations for endometrial cancer. For low-risk, surgically staged disease, observation alone is recommended based on consensus.

**Figure 5:**
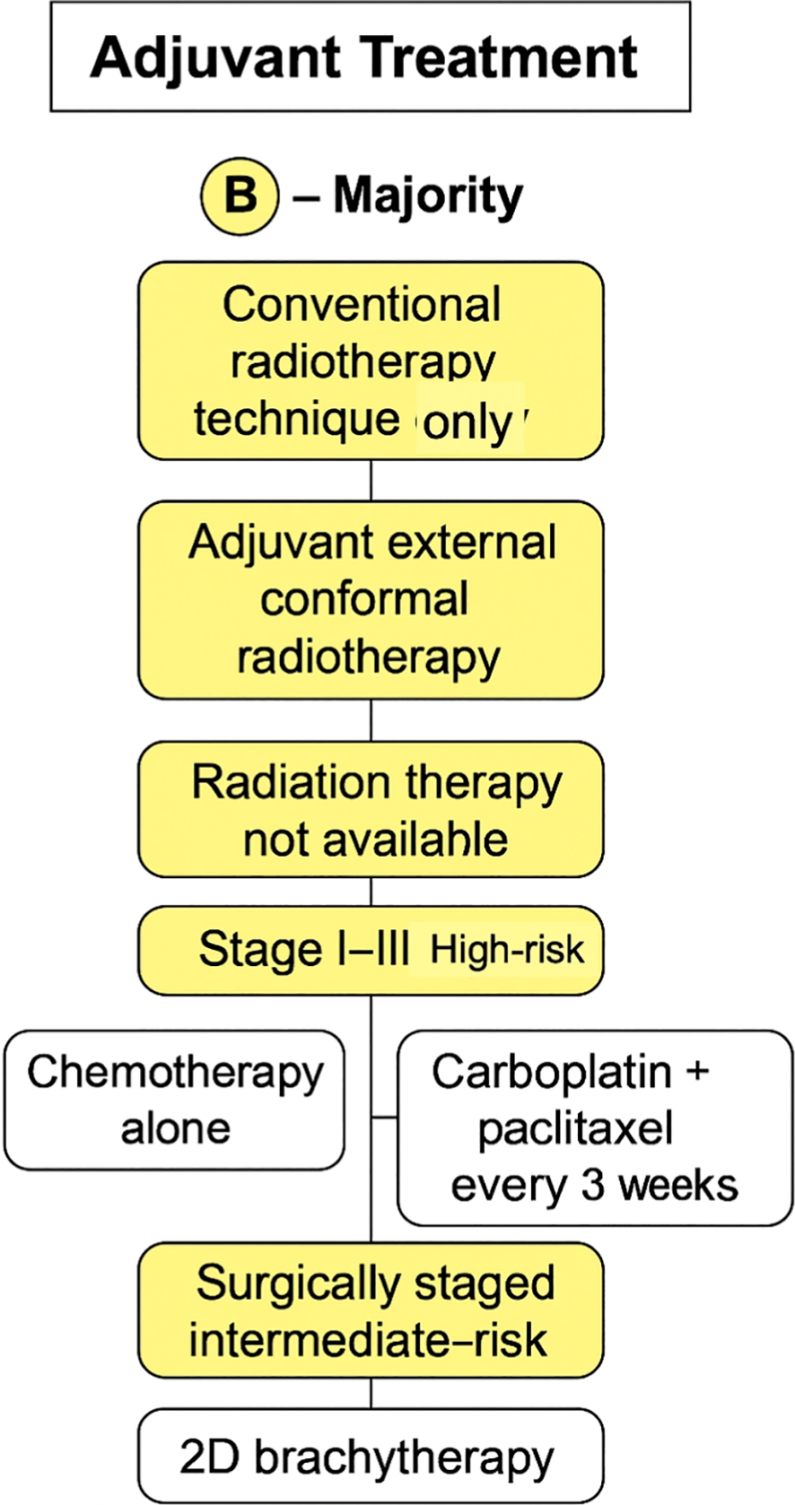
Adjuvant treatment recommendations based on majority vote. The algorithm outlines options for high-risk (stage I–III) and intermediate-risk disease, including external radiotherapy, chemotherapy (carboplatin plus paclitaxel), or brachytherapy depending on resource availability and surgical staging.

Finally, when those patients had no radiation therapy available, the majority (62%) decided on carboplatin and paclitaxel every 3 weeks as standard treatment. No consensus was achieved for how many treatment cycles to be given with opinions divided between four and six cycles. **Figures 4**, **5** provides a concise summary synthesizing the recommendations presented in this article

Recommendation by consensus vote:

- For low-risk endometrial carcinoma, surgically staged, the minimum acceptable adjuvant strategy is observation.

Recommendation by majority vote:

- In institutions where only conventional radiotherapy technique is available, patients with endometrial cancer can be treated with adjuvant external conformal radiotherapy, and the same suggestion was made in the scenario of primary treatment for those patients.
- For areas where radiation therapy is not available, for high-risk endometrial carcinoma, the minimum acceptable adjuvant strategy is chemotherapy alone.
- For surgically staged high-risk endometrial carcinoma, panelists agreed to offer adjuvant chemotherapy, if available.
- The minimum acceptable adjuvant brachytherapy technique for surgically staged intermediate-risk endometrial carcinoma is 2D.
- In areas without radiation therapy available, for patients with stage I-II high-risk endometrial carcinoma, the chemotherapy regimen of choice for adjuvant treatment was carboplatin and paclitaxel every 3 weeks.

### Section 5: first-line treatment of metastatic or locally advanced endometrial cancer

Forty-four questions were asked about locally advanced or metastatic endometrial cancer. Consensus was present for eight of those questions (**Supplementary Table 6**).

In institutions with only conventional radiotherapy, a strong majority of panelists (86%) supported treating locally advanced patients with primary external conformal radiotherapy, while 83% endorsed cobalt machines when this was the only available option for adjuvant external radiotherapy. Similarly, 83% agreed on administering a radiation boost for unresected nodes identified on imaging. 

Meanwhile, for advanced stages, when only conventional radiotherapy technique is available, a clear majority (90%) agree with offering treatment regardless of whether radiation is given with primary or adjuvant intent. For cobalt machines, 70% also favored the utilization. Chemotherapy with external radiation and brachytherapy was considered by 57% of panelists as the minimum acceptable adjuvant strategy for resected stage III and IV uterine endometrioid carcinoma. When radiation is not accessible, 65% settled for chemotherapy alone.

For half of the panelists (50%), the minimal acceptable chemotherapy regimen for adjuvant treatment in stage III is carboplatin and paclitaxel every 3 weeks, and 58% selected six cycles as a critical number. No results on the chemotherapy regimen were reached for stage IV endometrial cancer, but panelists agreed with also offering six cycles of treatment. Likewise, when offered monotherapy with carboplatin or carboplatin and paclitaxel, the panel was divided between chemotherapy regimens for when cisplatin was contraindicated and patients had significant comorbidities. Meanwhile, when taxanes were not available, no consensus was also reached deciding whether the best approach should comprise a combination of platinum regimens (platinum and 5- fluorouracil, platinum and cyclophosphamide, platinum and doxorubicin) or monotherapy (cisplatin or carboplatin).

Concerning stage IVA, no formal consensus was reached for the best treatment approach in areas with or without access to radiation. When radiation therapy was not available and specialized surgeons were not available 61% voted in favor of palliative chemotherapy. For patients who are stage IVB, panelists voted in favor of palliative chemotherapy as the treatment of choice, regardless of the availability of specialty oncology surgeons or radiotherapy.

In cases of recurrent platinum-sensitive endometrial cancer, the proposed salvage treatment that a higher proportion of panelists chose was chemotherapy re-exposure to carboplatin and paclitaxel every 3 weeks. As for patients with severe comorbidities, 43% voted for hormonal therapy for low-grade disease. For recurrent platinum- resistant/refractory, the most voted option for patients previously exposed to taxane was gemcitabine, for patients not exposed, then weekly paclitaxel was appointed. Also, regarding those patients when no clinical trial is available, the indication for best supportive care is made when performance status is > 2, unrelated to the patient’s line of treatment.

When questioned about appropriate treatment options for metastatic endometrial cancer from the WHO essential medicines list, three drugs were agreed upon consensus: aromatase inhibitors (94%), tamoxifen (89%), and progestins (85%).

**Figure 6:**
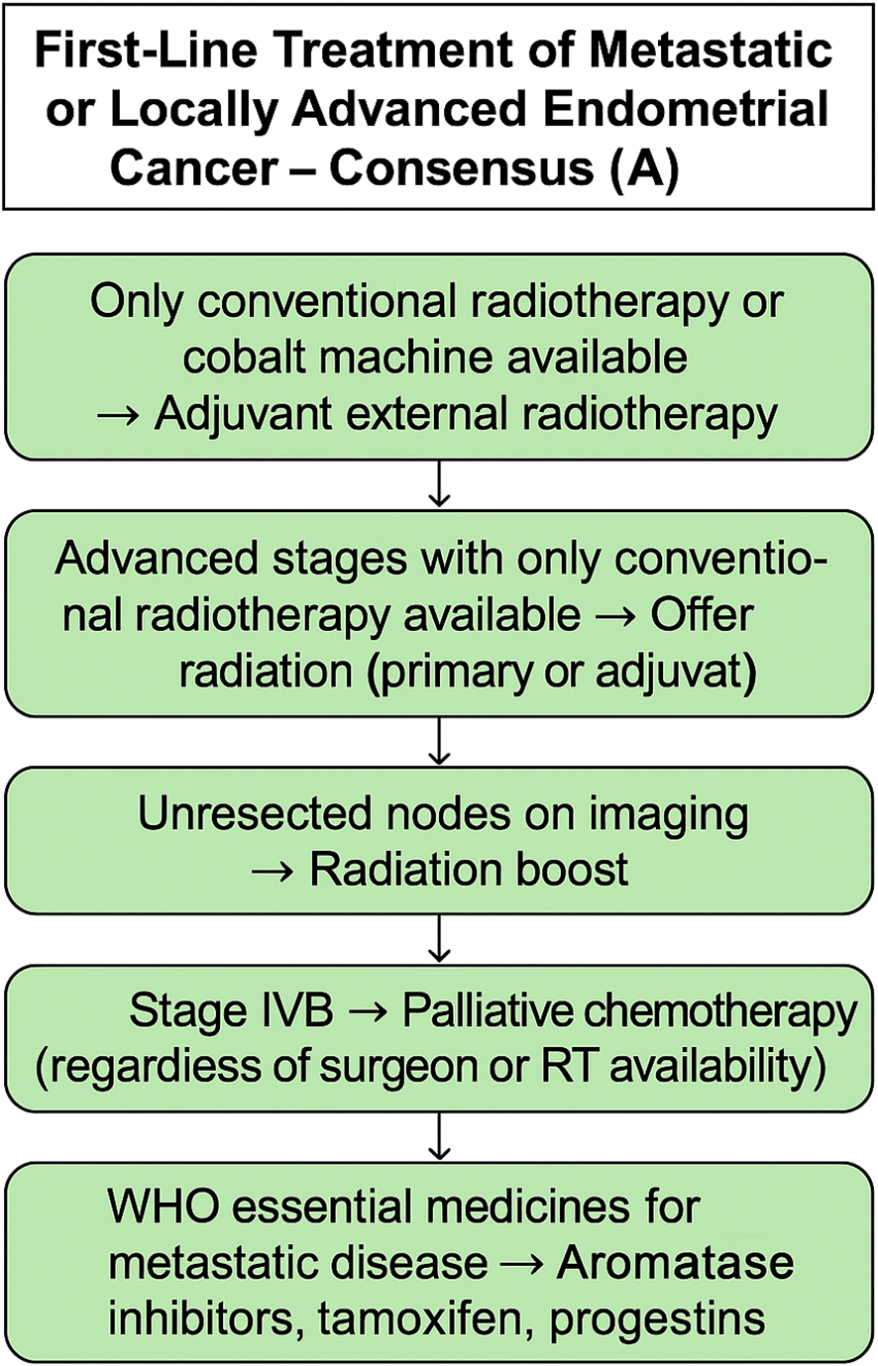
First-line treatment of metastatic or locally advanced endometrial cancer (consensus). Recommendations include radiotherapy when available, radiation boost for unresected nodes, and palliative chemotherapy for stage IVB disease, with consideration of WHO essential medicines in resource-limited settings.

**Figure 7:**
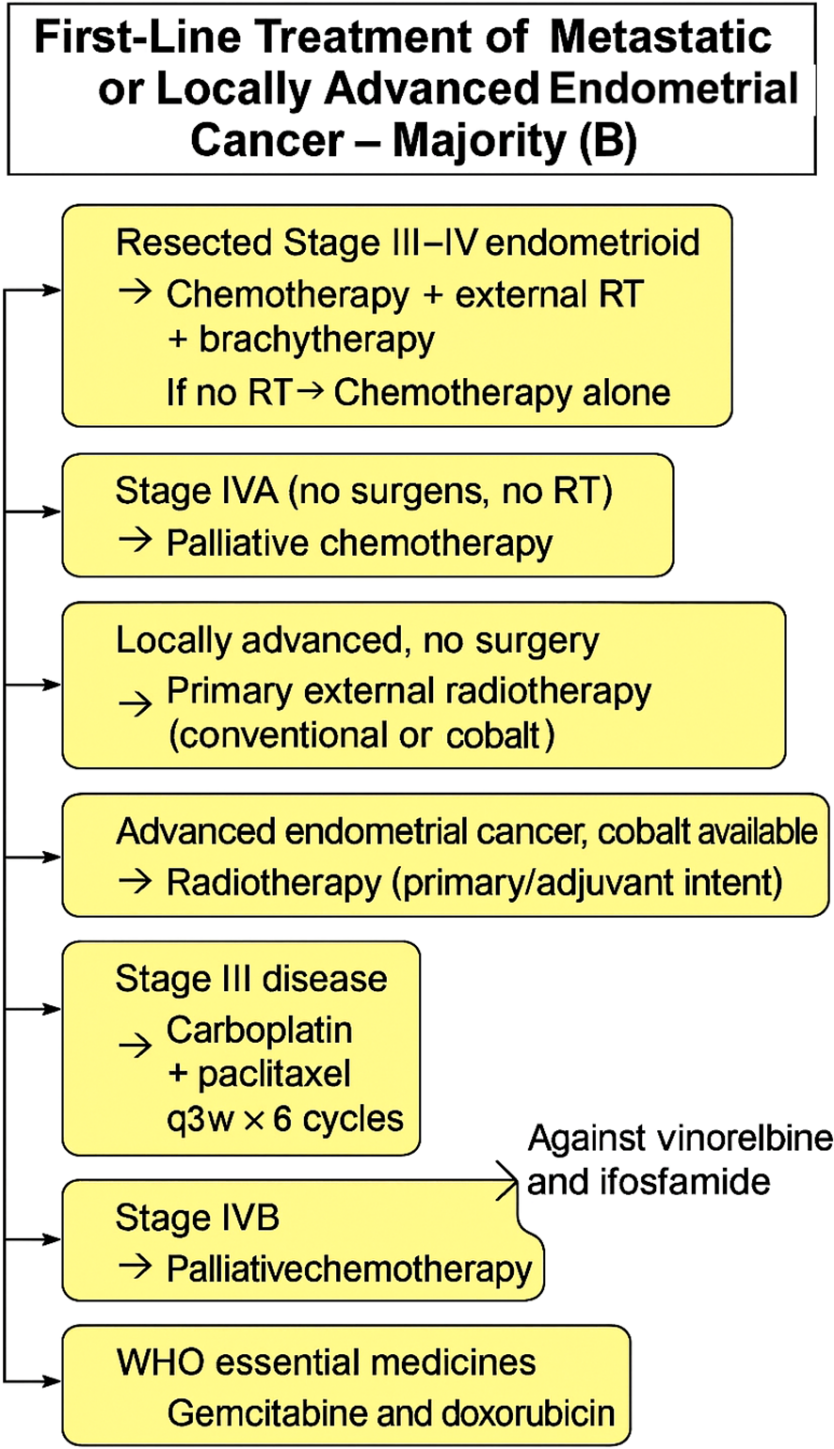
First-line treatment of metastatic or locally advanced endometrial cancer (majority vote). Management options include combined chemotherapy and radiotherapy for resected stage III–IV disease, chemotherapy alone when radiotherapy is unavailable, and palliative chemotherapy for stage IVB disease.

Majority voting was also achieved in favor of gemcitabine (63%), and doxorubicin (71%). There was also a majority vote against the use of vinorelbine (50%) and ifosfamide (69%). No decision was made for the best hormonal therapy, but a frequent vote favored progestins. **Figures 6**, **7** provides a concise summary synthesizing the recommendations presented in this article.

Recommendation by consensus vote:

- When only conventional radiotherapy or cobalt machine is available, patients with locally advanced endometrial cancer can be treated with adjuvant external radiotherapy.
- For advanced stages, when only conventional radiotherapy is available regardless of primary or adjuvant treatment, radiation should be offered.
- Patients with unresected nodes visualized on imaging should receive a radiation boost to achieve better local control.
- For patients with stage IVB endometrial cancer, the best treatment approach is palliative chemotherapy regardless of access to specialized surgeons and radiotherapy.
- Appropriate treatment options for metastatic endometrial cancer from the WHO essential medicines list are aromatase inhibitors, tamoxifen, and progestins.

Recommendation by majority vote:

- For resected stage III and IV uterine endometrioid carcinoma, minimal acceptable adjuvant treatment is chemotherapy, external radiation, and brachytherapy, when radiation is not available only offer chemotherapy alone.
- For stage IVA endometrial cancer in areas with no access to specialized oncology surgeons and radiation treatment should be palliative chemotherapy.
- When only a cobalt machine is available, and surgery is not an option, patients with locally advanced endometrial cancer can be treated with primary external radiotherapy.
- When conventional radiotherapy is available, and surgery is not an option, patients with locally advanced endometrial cancer can be treated with primary external radiotherapy.
- When only a cobalt machine is available, patients with advanced endometrial cancer should be treated either for primary or adjuvant intent when it is indicated.
- The minimal acceptable adjuvant treatment for stage III disease is carboplatin and paclitaxel every 3 weeks for a total of at least six cycles.
- For patients with stage IVB endometrial cancer, the best treatment approach is palliative chemotherapy regardless of access to specialized surgeons or radiotherapy.
- For stage IV endometrial and recurrent platinum-sensitive endometrial cancer, the minimum acceptable chemotherapy cycles are six.
- For platinum-resistant/refractory cancer, when no clinical trial is available, the indication for best supportive care is made when performance status is > 2, unrelated to the patient’s line of treatment.
- The appropriate treatment options for metastatic endometrial cancer, from the WHO essential medicines list are gemcitabine and doxorubicin. There was also a majority vote against the use of vinorelbine and ifosfamide.

### Section 6: recurrent endometrial cancer

Nine questions were asked about recurrent endometrial carcinoma. Consensus was present for two of those questions (**Supplementary Table 6**).

When recurrence in the vaginal area occurs, for patients previously treated with surgery, the minimum acceptable strategy in areas without trained surgeons and without radiotherapy is the indication of chemotherapy. Whereas in cases of pelvic lymph node recurrence for patients previously treated with surgery and radiation therapy with or without brachytherapy, when no oncology surgeon is available, the minimally acceptable option is also chemotherapy alone.

A majority vote was present for four of the nine questions.

**Figure 8:**
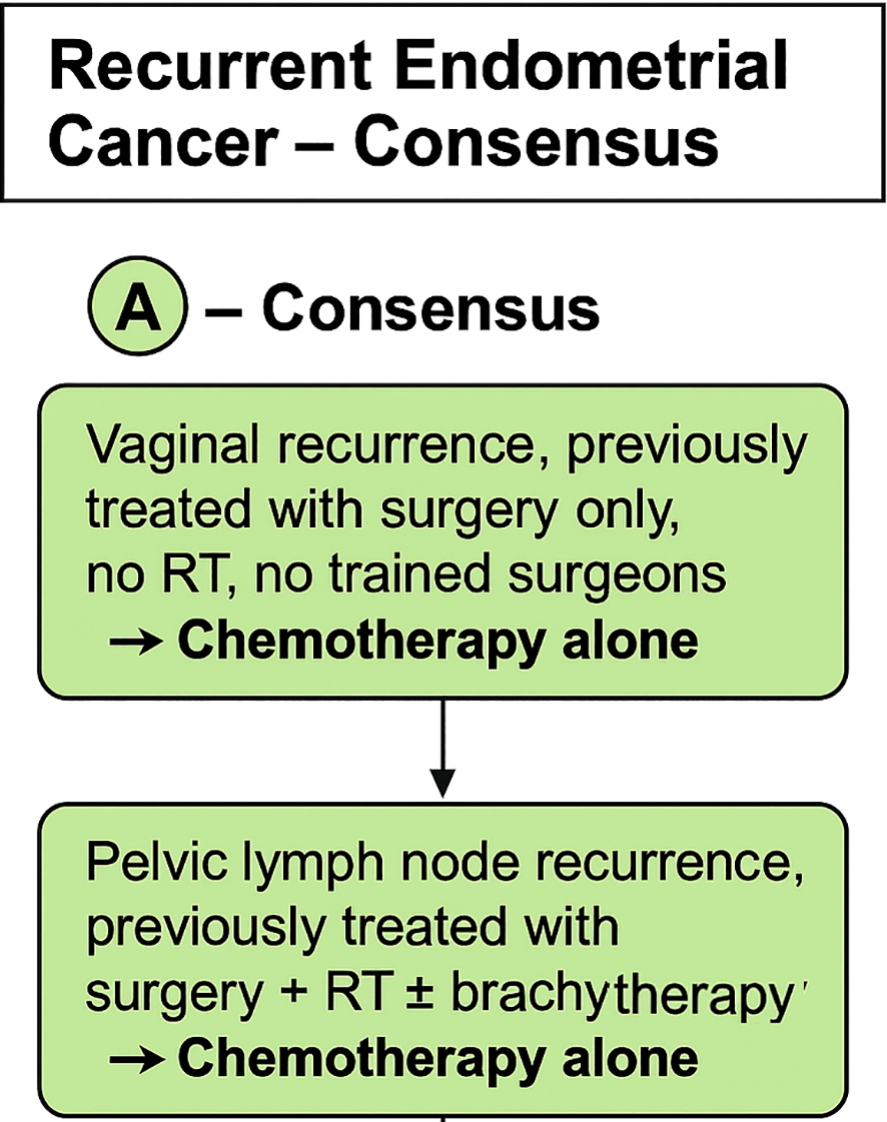
Management of recurrent endometrial cancer (consensus). For vaginal or pelvic nodal recurrence in previously treated patients without access to radiotherapy or trained surgeons, chemotherapy alone is recommended.

**Figure 9:**
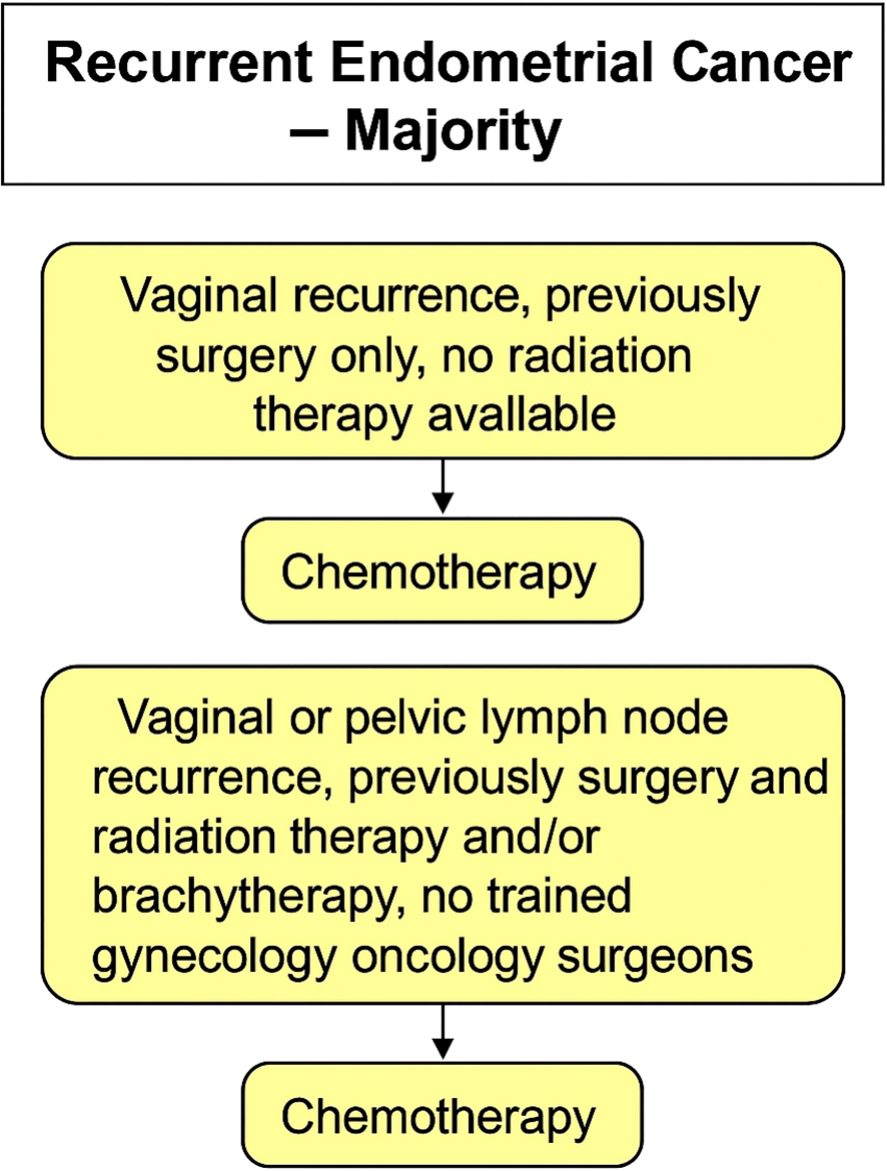
Management of recurrent endometrial cancer (majority vote). In cases of vaginal or pelvic recurrence with limited surgical or radiotherapy resources, chemotherapy is the preferred treatment option.

In vaginal recurrence treated only with surgery previously and where radiation therapy is not available, 70% of panelists considered salvaged surgery and chemotherapy to be the best treatment option. The minimum acceptable radical RT strategy for those patients who did not receive radiation after surgery is 2D external radiation therapy. In vaginal or pelvic lymph node recurrence patients previously treated with surgery and radiation therapy (external) and/or brachytherapy, in areas where surgeons do not have full training in gynecology oncology, 69% and 90%, respectively of the panelists agreed that the patient should undergo chemotherapy alone. **Figures 8**, **9** provides a concise summary synthesizing the recommendations presented in this article.

Recommendation by consensus vote:

- The minimum acceptable strategy for vaginal recurrence treated previously only with surgery in areas of severe resource limitations when radiation therapy is not available and when surgeons do not have full training in gynecology oncology is chemotherapy alone.
- Whereas for those patients with pelvic lymph node recurrence from endometrial cancer, treated previously with surgery and radiation therapy (external) with or without brachytherapy in areas where surgeons do not have full training in gynecology oncology the only approach should be chemotherapy alone.

Recommendation by majority vote:

- In vaginal recurrence treated previously only with surgery and where radiation therapy is not available, chemotherapy should be offered for those patients.
- In vaginal or pelvic lymph node recurrence treated previously with surgery and radiation therapy and/or brachytherapy with no trained gynecology oncology surgeons, chemotherapy should be offered.

## Discussion

Endometrial cancer is a challenging disease with rapidly evolving management in developed areas and supported by guidelines focused on when all the therapeutic tools are available. For low- and middle-income countries, the benefit of a guideline attempting to provide higher-quality recommendations is crucial. To our knowledge, this is the first consensus meeting aimed at this matter.

This consensus statement provides decision making guidance, adapted to areas where improvement in care is urgently needed. Multidisciplinary planning is encouraged, but recommendations were tailored to setting with limited availability of specialists, imaging and therapies. Given the low resources and limited tools, care does not always follow strict international consensus or high-level evidence; therefore, careful reading is advised, and the application of these recommendations is intended primarily for daily practice in areas with scarce resources. Importantly, the recommendations presented herein are consensus-based and derived from expert agreement rather than outcome-validated clinical trials. As such, they should be interpreted as pragmatic guidance reflecting current practice realities rather than evidence of superiority of one strategy over another. Even though a higher consensus for a more significant number of questions was appealing, consensus was reached for only eighteen of 109 (16.5%) questions presented to the panel, moreover, a majority vote was present for 43 (39.4%) additional questions. This likely reflects the heterogeneity in care delivery, infrastructure, and expertise across developing countries.

Regarding initial assessment, the consensus was achieved in the following cases: (1) for patients with stage III-IV endometrial cancer initial assessment should include a physical exam, pelvic and abdominal computed tomography, and chest x-ray; (2) in areas where there is no access to computed tomography reasoning was toward switching it to pelvic and abdominal ultrasound. Ultrasound remains an available resource with lower costs compared to other imaging methods and similar accuracy in endometrial cancer (6).

Considering surveillance, consensus was reached for the following question: (1) for stages III and IV after curative intent, optimal follow-up should be every 3 months in the first 2 years, afterward every 6 months until 5 years from treatment. Considerable practice variability exists toward surveillance among oncologists; the TOTEM trial attempted to refine follow-up strategies for low- and high-risk patients, demonstrating no improvement in early recurrence detection or overall survival with intensive surveillance. This highlights the need for better patient stratification based on relapse risk, which may directly influence healthcare costs (7).

In early-stage disease concerning surgical approach, a consensus was reached for the following questions: (1) in endometrioid grade 1 and 2, ECOG 2 or more and/or severe comorbidities in an area with untrained oncology surgeons the best single approach for patients should be total abdominal hysterectomy and bilateral salpingoophorectomy and in the case of no access to radiation therapy the same recommendation prevails; (2) in areas with limited resources, intraoperative gross examination it’s recommended after training in surgical staging.

There was no consensus regarding surgical management for non-specified histologic type, grade, or molecular subtype, nor for high-grade histologies, although a frequent approach was TAH, BSO, and pelvic lymph node sampling or dissection. In contrast, when adequate oncology surgical training was unavailable, the majority of panelists chose not to perform systematic lymphadenectomy, even when bulky suspicious lesions were present intraoperative. It is common knowledge, based on studies conducted in other gynecology malignancies, that surgical training is directly associated with better prognosis and fewer complications when there is a need for systematic lymphadenectomy (8). For endometrial carcinoma, the role of the procedure for therapeutic reasons is dubious, but for prognostic significance and assisting with decision for adjuvant treatment, the contribution is unquestionable (9).

As for adjuvant treatment in early-stage disease, consensus was reached that observation alone is appropriate for surgically staged low-risk endometrial carcinoma. Prospective data increasingly support a risk-adapted approach to adjuvant therapy incorporating molecular classification. The phase III PORTEC-4a trial evaluated a molecular-integrated risk profile to guide adjuvant treatment decisions in early-stage endometrial cancer, comparing standard adjuvant radiotherapy with molecular profile–based management. This strategy enabled treatment de-escalation in patients with favorable molecular features, such as POLE-mutated tumors, and escalation in those with unfavorable profiles, thereby reducing overtreatment without compromising oncologic outcomes. Although the comprehensive molecular stratification applied in PORTEC-4a remains difficult to implement in low-resource settings, its results reinforce the principle that adjuvant therapy intensity should be driven by individualized recurrence risk rather than uniform clinicopathologic criteria alone.

Adjuvant therapy decisions in intermediate- and high-risk disease remain particularly complex, as this is the subgroup in which molecular information is most likely to alter treatment recommendations. In resource-limited settings, widespread molecular profiling is constrained by cost, limited access, and shortages of specialized pathology expertise. A pragmatic, stepwise implementation of molecular classification may therefore be most feasible, prioritizing testing in clinically equivocal or high-impact scenarios, particularly among intermediate-risk patients. Practical approaches include centralized or referral-based testing, regional pathology hubs, and selective use of immunohistochemistry as surrogates for molecular profiling. Specifically, mismatch repair protein immunohistochemistry to identify MMR-deficient tumors, p53 immunohistochemistry to detect copy-number–high carcinomas, and targeted POLE mutation testing in selected cases may represent achievable initial steps. Where molecular classification is not available, adjuvant treatment decisions should continue to rely on traditional clinicopathologic factors, with molecular data incorporated when feasible to refine risk stratification and guide therapy (10, 11).For intermediate-risk patients, adjuvant brachytherapy provides excellent local control and high survival rates, similar to those offered by adjuvant external radiation therapy, as shown in large randomized trials (12, 13). Nevertheless, there are numerous geographic and economic barriers to providing brachytherapy for cancer patients in limited resource areas (14). That fact is probably one of the reasons why only 33% of panelists chose brachytherapy as the treatment of choice for this subgroup of patients. When no radiation was available, the decision was dichotomized into two main options: chemotherapy or observation. As far as the minimal accepted adjuvant brachytherapy technique, 63% of panelists designated 2D.

For surgically staged high-risk patients, the panel generally indicated chemotherapy plus or minus radiation therapy when available, which can be explained based on the costs and availability of chemotherapy even in underserved areas. The potential benefit of adding chemotherapy to decrease the risk of recurrence has been evaluated in three relevant phase III trials (GOG-258, GOG 249 and PORTEC-3), which included intermediate-risk and high-risk patients.

The phase III trial GOG-249 assessed the impact of recurrence-free survival (RFS) between pelvic radiotherapy or brachytherapy followed by three cycles of paclitaxel and carboplatin, the 5-year RFS and overall survival (OS) showed no differences between both arms. In GOG-258, patients were randomized to pelvic radiotherapy with concurrent and adjuvant chemotherapy or chemotherapy alone with once again no differences in RFS and OS. Still, there were notably more recurrences in vaginal and para- aortic and/or pelvic recurrences in women treated with chemotherapy alone. Moreover, an updated follow-up of the PORTEC-3 trial, which evaluated the role of chemotherapy during and after radiation versus pelvic radiation alone, showed a 5% benefit in OS and a 7% benefit in failure-free survival for the concurrent plus adjuvant chemotherapy group, compared with radiation alone. Taking into consideration the combined results of these trials the decision-making should be individualized on a case-by-case basis, weighing the benefits and potential toxicities of the treatment and also the availability of techniques (15–17).

For stages III and IV multimodality treatment (chemotherapy, external radiation, and brachytherapy) was the option of choice. When determining which chemotherapy regime was most appropriate for adjuvant treatment, the decision was once again divided between regimens involving carboplatin and paclitaxel every 3 weeks or cisplatin in combination with paclitaxel for four to six cycles.

The best chemotherapy regimen was addressed In JGOG 2043, a phase III randomized clinical trial that compared regimens of carboplatin and paclitaxel, cisplatin and docetaxel or cisplatin and doxorubicin with no significant difference of survival among patients receiving any regimen, but better tolerability and side effects profile for carboplatin with paclitaxel, a regimen with convenient dosage schedule, who does not require growth factor support and it is well tolerated. All of these factors in areas with limited resources are also taken into consideration when deciding on treatment. Unfortunately, established treatment modalities widely available for higher-income countries, can be challenging to administer in low-income settings, as the cost and availability of chemotherapy drugs vary nationwide (18).

There was no consensus on the questions related to locally advanced disease (stage IVA) and treatment recommendation with the most significant proportion of votes typically involving neoadjuvant chemotherapy followed by surgery. This is the management for some centers where the waiting list for surgery precludes upfront surgery and favors a treatment as a bridge to proceed with surgery later on. Data suggests that locally advanced/bulky disease comprising stage III and IV maximal cytoreduction should be considered only if macroscopic complete resection is feasible, with acceptable morbidity (19, 20). When radiotherapy and specialized oncology surgeons were not available, the majority of panelists (61%) chose only to pursue palliative chemotherapy as the treatment of choice. A justifiable choice with limited resources, reinforced by the standard front-line treatment with chemotherapy regimen providing a 47% overall response rate in metastatic disease as noted in GOG 209 (21).

The only consensus reached in this setting was for the following question:

(1) when only conventional radiotherapy or cobalt machine is available, patients with locally advanced endometrial cancer can be treated with adjuvant external radiotherapy.

For metastatic patients, consensus was reached on the following matters: (1) For advanced stages when only conventional radiotherapy is available, radiation should be offered; (2) patients with unresected nodes that were visualized on imaging should be referred to radiation boost to achieve better local control; (3) patients with stage IVB, regardless of access to surgeons or radiotherapy should be offered palliative chemotherapy; (4) appropriate treatment options for metastatic endometrial cancer are aromatase inhibitors, tamoxifen and progestins.

Treatments based on cobalt units rely on unsophisticated techniques but have the advantage of requiring reduced maintenance, running costs, and downtime compared to linear accelerators.

Regarding recurrent endometrial cancer, a consensus was reached for the following questions: (1) for vaginal recurrence previously treated with surgery previously, in areas where radiation is not available and there are no trained surgeons, the acceptable management is chemotherapy only; (2) whereas for patients with pelvic lymph node recurrence treated previously with surgery and radiation, in areas with lack of specialized surgeons, the approach should also be chemotherapy alone.

When there is limited access to radiation techniques, notwithstanding offering for adjuvant or primary purpose of treatment in locally advanced cancer, panelists reasonably argued in favor of conventional radiation or cobalt machines radiation whichever was available at the service. Although the feasibility and dosimetric advantages of pelvic intensity modulated radiation therapy (IMRT) are well documented and demonstrate decreased volumes of bone marrow, bladder, bowel, and rectum receiving clinically significant doses of RT, in countries facing resource limitations, the reality of access to contemporary radiation therapy techniques is often a barrier (22, 23). Treatments based on cobalt units rely on unsophisticated techniques but have the advantage of requiring reduced maintenance, running costs, and downtime compared to linear accelerators (24). When comparing the efficacy and toxicities of intensity- modulated radiotherapy (IMRT) with three-dimensional (3D) conformal radiotherapy or 2D conventional radiotherapy, a phase III study performed in patients with cervical and endometrial cancer demonstrated equivalent efficacy, although IMRT significantly reduced toxicities (25). In regards to cost-effectiveness, IMRT is associated with reduced late overall toxicity compared to 3D and it becomes more cost-effective over time because of that (26).

On the subject of first-line treatment for advanced disease, almost half of the panelists appointed carboplatin and paclitaxel every 3 weeks as the regimen of choice, an option endorsed by the phase III clinical trial that established the non-inferiority of paclitaxel and carboplatin to cisplatin, doxorubicin, and paclitaxel (TAP), achieving a 40-50% overall response rate, median PFS of 14 months and OS of 32 months with more favo rable toxicity profile (27). When patients faced no access to taxanes, possibilities included a combination of platinum and 5-fluorouracil or platinum and doxorubicin. The last regimen was standard of care for many years until a phase III trial comparing it to taxane and platinum was suggested as a reasonable alternative with fewer toxicities. In contrast, the alternative with 5-fluorouracil is less explored in endometrial cancer. ( 28).

When possible, standard Western treatment regimens should be the first treatment of choice, but when faced with limitations, the alternative solution should approach the most accessible, effective, and affordable option available.

This consensus meeting is, to the best of our knowledge, the first attempt to provide recommendations for early, locally advanced, and recurrent or metastatic endometrial carcinoma, involving specialists from many countries facing severe limitations in medical access. In routine practice, most institutions strive to adhere to international guidelines such as those from the National Comprehensive Cancer Network (NCCN), ESMO, and others. However, given the increasing complexity of endometrial cancer management following the incorporation of molecular classification and new treatment options, it is of utmost importance to provide pragmatic guidance that reflects the realities of healthcare systems facing severe resource constraints.

In this context, the present consensus represents an initial step toward harmonizing care in resource-limited settings. Future directions should include implementation studies and real-world validation to assess the feasibility, adherence, clinical outcomes, and cost-effectiveness of these recommendations across diverse healthcare environments. Such efforts will be essential to refine guidance, inform policy development, and support iterative updates as resources and access to molecular diagnostics continue to evolve.

## Data Availability

The original contributions presented in the study are included in the article/**Supplementary Material**. Further inquiries can be directed to the corresponding authors.

## References

[1] Bray F . Global cancer statistics 2022: GLOBOCAN estimates of incidence and mortality worldwide for 36 cancers in 185 countries. CA Cancer J Clin. (2024). doi: 10.3322/caac.21834, PMID: 38572751

[2] Yang L . Time trend of global uterine cancer burden: an age-period-cohort analysis from 1990 to 2019 and predictions in a 25-year period. BMC Women’s Health. (2023) 23:384. doi: 10.1186/s12905-023-02535-5, PMID: 37480027 PMC10362563

[3] Anakwenze , Ewongwo A ,Onyewadume L , Oyekan A , Chigbo CO , Valle L . A systematic review of endometrial cancer clinical research in Africa. Infect Agent Cancer. (2024) 19(1):2. doi: 10.1186/s13027-023-00563-2, PMID: 38217018 PMC10787484

[4] Mattes MD . Overcoming barriers to radiation oncology access in low-resource settings in the United States. Adv Radiat Oncol. (2021) 6:100802. doi: 10.1016/j.adro.2021.100802, PMID: 34693080 PMC8515237

[5] Restaino . Management of patients diagnosed with endometrial cancer: comparison of guidelines. Cancers (Basel). (2023) 15:1091. doi: 10.3390/cancers15041091, PMID: 36831434 PMC9954548

[6] Wong M . A prospective comparison of the diagnostic accuracies of ultrasound and magnetic resonance imaging in preoperative staging of endometrial cancer. J Gynecol Oncol. (2022) 33:e22. doi: 10.3802/jgo.2022.33.e22, PMID: 35128854 PMC8899878

[7] Zola P . TOTEM collaborative group Effectiveness of intensive versus minimalist follow-up regimen on survival in patients with endometrial cancer (TOTEM study): A randomized, pragmatic, parallel group, multicenter trial. J Clin Oncol. (2022) 40:3817–27. doi: 10.1200/JCO.22.00471, PMID: 35858170

[8] duBoisA . Role of surgical outcome as prognostic factor in advanced epithelial ovarian cancer: a combined exploratory analysis of 3 prospectively randomized phase 3 multicenter trials: By the arbeitsgemeinschaft gynaekologische onkologie studiengruppe ovarialkarzinom (AGO-OVAR) and the groupe d’Investigateurs nationaux pour les etudes des cancers de l’Ovaire (GINECO). Cancer. (2009) 115:1234–44. doi: 10.1002/cncr.24149, PMID: 19189349

[9] Kitchener H . Efficacy of systematic pelvic lymphadenectomy in endometrial cancer (MRC ASTEC trial): a randomised study. Lancet. (2009) 373:125–36. doi: 10.1016/S0140-6736(08)61766-3, PMID: 19070889 PMC2646126

[10] Concin N . ESGO/ESTRO/ESP guidelines for the management of patients with endometrial carcinoma. Int J Gynecological Cancer. (2021) 31:12 –39. doi: 10.1136/ijgc-2020-002230, PMID: 33397713

[11] van den Heerik ASVM . Molecular profile-based adjuvant treatment for women with high-intermediate risk endometrial cancer (PORTEC-4a): results of a randomised, open-label, phase 3, multicentre, non-inferiority trial. Lancet Oncol. (2026) 27:23–35. doi: 10.1016/S1470-2045(25)00612-6, PMID: 41449145

[12] Barney BM . The role of vaginal brachytherapy in the treatment of surgical stage I papillary serous or clear cell endometrial cancer. Int J Radiat Oncol Biol Phys. (2013) 85:109–15. doi: 10.1016/j.ijrobp.2012.03.011, PMID: 22543202

[13] Sorbe B . External pelvic and vaginal irradiation versus vaginal irradiation alone as postoperative therapy in medium-risk endometrial carcinoma—a prospective randomized study. Int J Radiat Oncol Biol Phys. (2012) 82:1249–55. doi: 10.1016/j.ijrobp.2011.04.014, PMID: 21676554

[14] Grover S . The unique issues with brachytherapy in low- and middle-income countries. Semin Radiat Oncol. (2017) 27:136–42. doi: 10.1016/j.semradonc.2016.11.005, PMID: 28325239 PMC6661890

[15] Matei D . Adjuvant chemotherapy plus radiation for locally advanced endometrial cancer. N Engl J Med. (2019) 380:2317–26. doi: 10.1056/NEJMoa1813181, PMID: 31189035 PMC6948006

[16] de Boer SM PORTEC Study Group . Adjuvant chemoradiotherapy versus radiotherapy alone in women with high-risk endometrial cancer (PORTEC-3): patterns of recurrence and *post-hoc* survival analysis of a randomised phase 3 trial. . Lancet Oncol. (2019) 20:1273–85. doi: 10.1016/S1470-2045(19)30395-X, PMID: 31345626 PMC6722042

[17] Randall ME . Phase III trial: adjuvant pelvic radiation therapy versus vaginal brachytherapy plus paclitaxel/carboplatin in high-intermediate and high-risk early stage endometrial cancer. J Clin Oncol. (2019) 37:1810–8. doi: 10.1200/JCO.18.01575, PMID: 30995174 PMC6804858

[18] Basile S . Gynecological cancers in developing countries: the challenge of chemotherapy in low-resources settings. Int J Gynecol Cancer. (2006) 16:1491–7. doi: 10.1111/j.1525-1438.2006.00619.x, PMID: 16884356

[19] Barlin JN . Cytoreductive surgery for advanced or recurrent endometrial cancer: a meta-analysis. Gynecol Oncol. (2010) 118:14–1. doi: 10.1016/j.ygyno.2010.04.005, PMID: 20434198

[20] Cirik DA . Stage IVb endometrial cancer confined to the abdomen: is chemotherapy superior to radiotherapy? Eur J Gynaecol Oncol. (2016) 37:226–31., PMID: 27172750

[21] Miller DS . Carboplatin and paclitaxel for advanced endometrial cancer: final overall survival and adverse event analysis of a phase III trial (NRG oncology/GOG0209). J Clin Oncol. (2020) 38:3841–50. doi: 10.1200/JCO.20.01076, PMID: 33078978 PMC7676887

[22] Mell LK . Dosimetric comparison of bone marrow-sparing intensity-modulated radiotherapy versus conventional techniques for treatment of cervical cancer. Int J Radiat Oncol Biol Phys. (2008) 71:1504–10. doi: 10.1016/j.ijrobp.2008.04.046, PMID: 18640499

[23] Harkenrider MM . Radiation therapy for endometrial cancer: an american society for radiation oncology clinical practice guideline. Pract Radiat Oncol. (2023) 13:41–65. doi: 10.1016/j.prro.2022.09.002, PMID: 36280107

[24] Adams EJ . A comparison between cobalt and linear accelerator-based treatment plans for conformal and intensity-modulated radiotherapy. Br J Radiol. (2008) 81:304–10. doi: 10.1259/bjr/77023750, PMID: 18250119

[25] Eung AR . Intensity-modulated radiation therapy reduces patient-reported chronic toxicity compared with conventional pelvic radiation therapy: updated results of a phase III trial. J Clin Oncol. (2022) 40:3115–9. doi: 10.1200/JCO.21.02831, PMID: 35960897 PMC9851703

[26] Chen LA . Toxicity and cost-effectiveness analysis of intensity modulated radiation therapy versus 3-dimensional conformal radiation therapy for postoperative treatment of gynecologic cancers. Gynecol Oncol. (2015) 136:521–8. doi: 10.1016/j.ygyno.2014.12.039, PMID: 25562668

[27] Miller DS . Carboplatin and paclitaxel for advanced endometrial cancer: final overall survival and adverse event analysis of a phase III trial (NRG Oncology/GOG0209). J Clin Oncol. (2020) 38:3841–50. doi: 10.1200/JCO.20.01076, PMID: 33078978 PMC7676887

[28] Nomura H Japanese Gynecologic Oncology Group . Effect of Taxane Plus Platinum Regimens vs Doxorubicin Plus Cisplatin as Adjuvant Chemotherapy for Endometrial Cancer at a High Risk of Progression: A Randomized Clinical Trial. JAMA Oncol. (2019) 5:833–40. doi: 10.1001/jamaoncol.2019.0001, PMID: 30896757 PMC6567838

