# Supplementary material for: Recommendations for the treatment of endometrial cancer in settings with limited resources: report from the International Gynecological Cancer Society consensus meeting
Source: Front Oncol. 2026 Mar 6;16:1676000. doi: 10.3389/fonc.2026.1676000 (PMC13002782; doi:10.3389/fonc.2026.1676000)
Supplement: Supplementary file 1 [file DataSheet1.docx]

**Supplementary Materials for “”“GLOBAL CONSENSUS FOR THE SCREENING, DIAGNOSIS, STAGING, TREATMENT, AND SURVEILLANCE OF ENDOMETRIAL CANCER IN AREAS OF SIGNIFICANT RESOURCE LIMITATIONS: REPORT FROM THE INTERNATIONAL GYNECOLOGICAL CANCER SOCIETY CONSENSUS MEETING ‘ ‘’**

**Supplementary Table 1: List of 10 countries represented by the panel**

Argentina, Brazil, Chile, Colombia, Guatemala, Israel, Lebanon, Mexico, Omã, Poland.

Members of the panel by subspecialty:

Oncologists: Fernando Cotait Maluf¹, Francinne T Tostes¹, Henrique Alkalay Helber¹, Graziela Z. Dal Molin², Angélica Nogueira Rodrigues⁵, Andreia Cristina de Melo⁸, Eduardo Paulino⁸, Glauco Baiocchi Neto⁹, Leandro Santos de Araujo Resende¹⁰, Michelle Samora de Almeida¹¹, Diocesio Pinto¹², Gabriel Rendon²⁰, Jimena Meymar²⁶

Gynecologic-Oncology-Surgeons: David Isla Ortiz⁴, Renato Moretti Marques¹, Bruno Roberto Braga Azevedo³, Agnaldo Lopes Silva Filho⁵, Reitan Ribeiro⁶, Georgia Fontes Cintra⁷, Gustavo Guitmann⁸, Julio Lau de la Vega¹³, Florencia Noll¹⁴, Juliana Rodriguez¹⁵, Fabio Fin¹⁶, David Cantu⁴, Andre Lopes⁹, Miguel Enrique Matute Correa¹⁷, Joseph S. Ng¹⁸, Daniel Sanabria¹⁹ Fernando Heredia²¹, Myriam Beatriz Perotta Mussi²³ Marcin Stanisław Bobiński²⁴, Milagros Perez Quintanilla²⁵, David Atallah²⁷

Radiation oncologists: Ligia Casao Arteaga¹, Juliana Karassawa Helito¹, Ana Paula E. Galerani Lopes²²

**Supplementary Table 2.** Questions related to initial assessment of endometrial cancer.


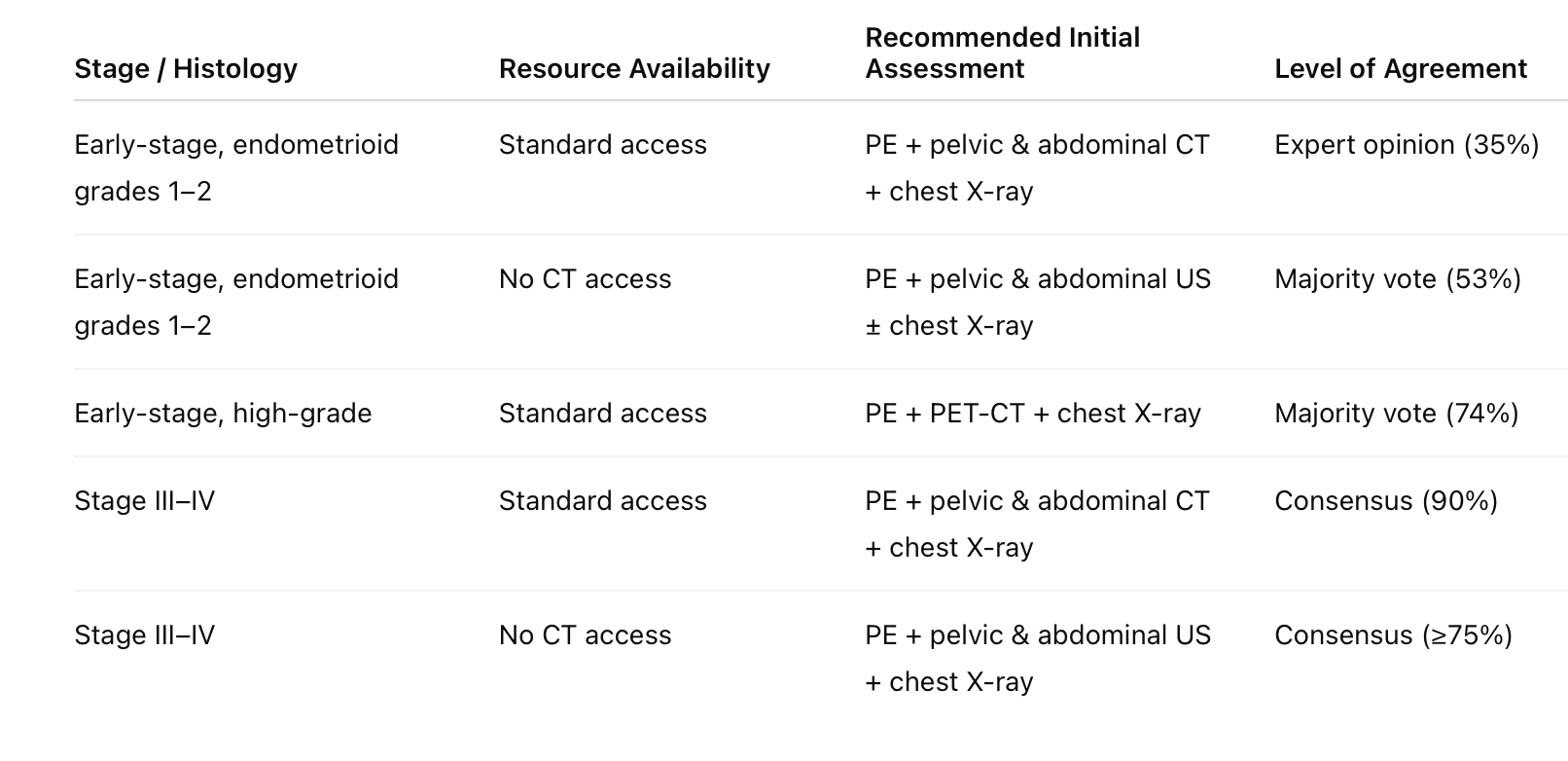


| Questions | **Responses** (%) | | | | | | |
| --- | --- | --- | --- | --- | --- | --- | --- |
| How should patients with early stage endometrioid grades 1 and 2 endometrial cancer be initially assessed in an area of severe limited resources? | Only physical exam | Physical exam + Pelvic and abdominal ultrasound | Physical exam + pelvic and abdominal ultrasound + chest X-Ray | Physical exam + Pelvic and abdominal computed tomography | Physical exam + Pelvic and abdominal computed tomography + Chest X-Ray | Abstain | Unqualified to answer |
|  | 5% | 20% | 35% | 5% | 35% | - | - |
| How should patients with early stage endometrioid grades 1 and 2 endometrial cancer be initially assessed in an area of severe limited resources if computed tomography is not available? | Only physical exam | Physical exam and pelvic and abdominal ultrasound | Physical exam + Pelvic and abdominal ultrasound + Chest X-Ray | Abstain | Unqualified to answer |  |  |
|  | 10% | 37% | 37% | - | - |  |  |
| How should patients with early stage high grade histologies be initially assessed in an area of severe limited resources? | Only physical exam | Physical exam and pelvic and abdominal ultrasound | Physical exam + Pelvic and abdominal ultrasound + Chest X-Ray | Physical exam + Pelvic and abdominal computed tomography | Physical exam + Pelvic and abdominal computed tomography + Chest X-Ray | Abstain | Unqualified to answer |
|  | 5% | - | 16% | 5% | 74% | - | - |
| How should patients with early stage high grade histologies be initially assessed in an area of severe limited resources if computed tomography is not available? | Only physical exam | Physical exam and pelvic and abdominal ultrasound | Physical exam + Pelvic and abdominal ultrasound + Chest X-Ray | Abstain | Unqualified to answer |  |  |
|  | 10% | 19% | 71% | - | - |  |  |
| How should patients with stage III-IV endometrial cancer be initially assessed in an area of severe limited resources? | Only physical exam | Physical exam and pelvic and abdominal ultrasound | Physical exam + Pelvic and abdominal ultrasound + Chest X-Ray | Physical exam + Pelvic and abdominal computed tomography | Physical exam + Pelvic and abdominal computed tomography + Chest X-Ray | Abstain | Unqualified to answer |
|  | 5% | - | - | 5% | 90% | - | - |
| How should patients with stage III-IV endometrial cancer be initially assessed in an area of severe limited resources if computed tomography is not available? | Only physical exam | Physical exam and pelvic and abdominal ultrasound | Physical exam + Pelvic and abdominal ultrasound + Chest X-Ray | Abstain | Unqualified to answer |  |  |
|  | 5% | 10% | 85% | - | - |  |  |

**Supplementary Table 3.** Questions related to surveillance of endometrial cancer.

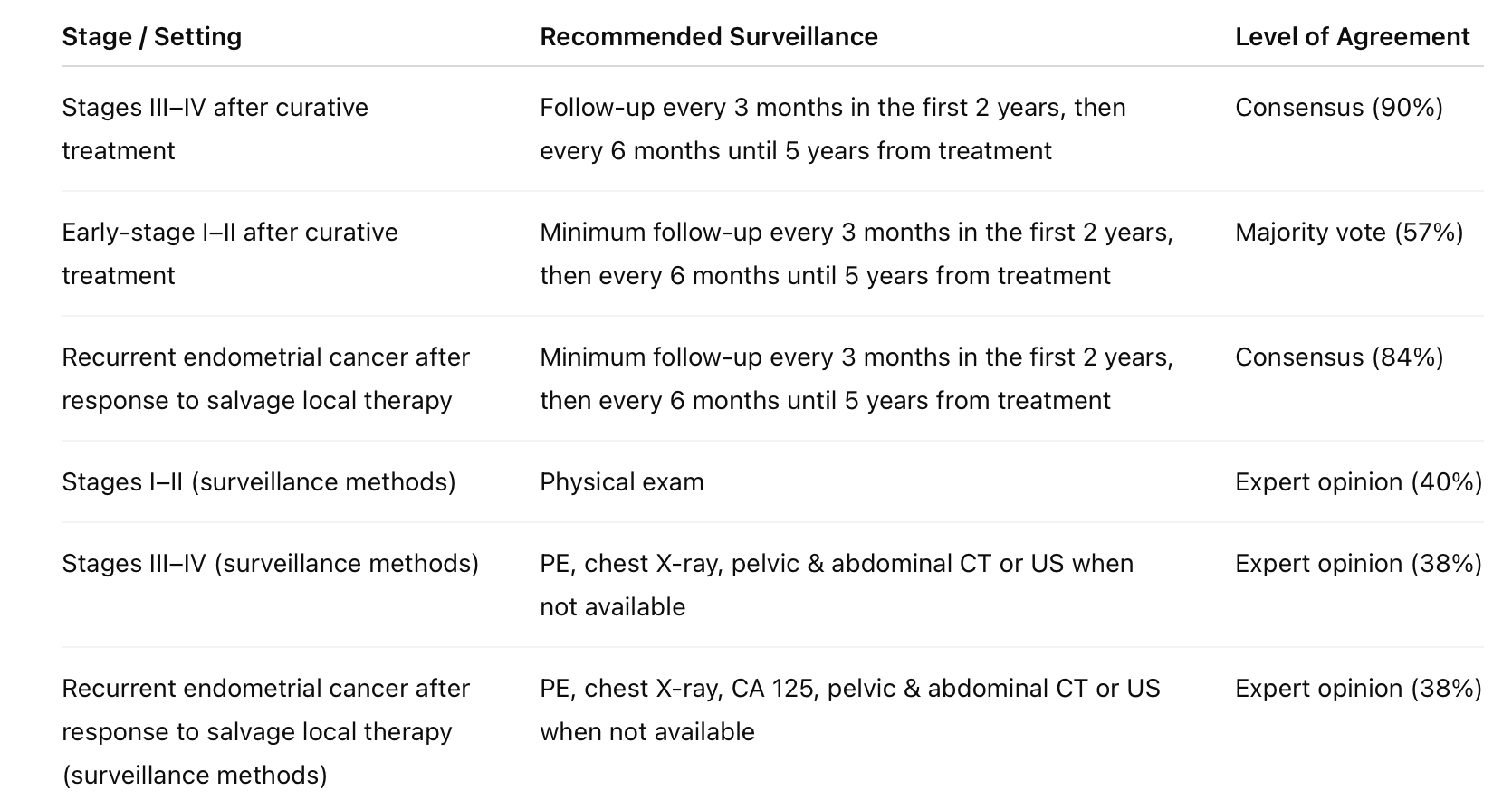


| Questions | **Responses** (%) | | | | | | |
| --- | --- | --- | --- | --- | --- | --- | --- |
| What’s the minimum acceptable frequency of follow-up for stage I-II endometrial cancer patients after curative treatment in an area with severe resources limitations? | Every 3 months in the first 2 years, after that, every six months until 5 years from the treatment | Every 6 months until 5 years from the treatment | Every 6 months in the first 2 years, adter that, annualy until 5 years from the treatment | None until the presence of symptoms | Abstain | Unqualified to answer | Annually until 5 years from the treatment |
|  | 57% | 33% | 10% | - | - | - | - |
| What is the minimum acceptable tools for follow up in patients with stage I-II disease after curative treatment in an area with severe limited resources? | Clinical examination only | CA 125 | Clinical examination + CA 125 | Pelvic and abdominal ultrasound and chest X-Ray + CA 125 + Physical exam | Pelvic and abdominal ultrasound and chest X-Ray + physical exam | Pelvic and abdominal computed tomography and chest X-Ray + Physical exam + CA 125 | Pelvic and abdominal computed tomography and chest X-Ray + CA 125+ Physical exam + vault cytology |
|  | 40% | - | 10% | 5% | 15% | 15% | 10% |
| What is the minimum acceptable tools for follow up in patients with stage I-II disease after curative treatment in an area with severe limited resources if computed tomography is not available? | Only physical exam | Physical exam + CA 125 | Physical exam + Pelvic and abdominal ultrasound + Chest X-Ray | Physical exam + Pelvic and abdominal ultrasound + Chest X-Ray +CA 125 | Physical exam + Pelvic and abdominal ultrasound + Chest X-Ray +CA 125 + vault cytology | CA 125 |  |
|  | 48% | 9% | 29% | 9% | 5% | - |  |
| What’s the minimum acceptable frequency of follow-up for stage III-IV endometrial cancer patients after curative treatment in an area with severe resources limitations? | Every 3 months in the first 2 years, after that, every six months until 5 years from the treatment | Every 6 months until 5 years from the treatment | Every 6 months in the first 2 years, adter that, annualy until 5 years from the treatment | None until the presence of symptoms | Abstain | Unqualified to answer | Annually until 5 years from the treatment |
|  | 90% | 5% | 5% | - | - | - | - |
| What is the minimum acceptable tools for follow up in patients with stage III-IV disease after curative treatment in an area with severe limited resources? | Clinical examination only | CA 125 | Clinical examination + CA 125 | Pelvic and abdominal ultrasound and chest X-Ray + Physical exam | Pelvic and abdominal ultrasound and chest X-Ray + Physical exam + CA 125 | Pelvic and abdominal computed tomography and chest X-Ray + CA 125 + Physical exam | Pelvic and abdominal computed tomography and chest X-Ray + CA 125 + Physical exam + Vault cytology |
|  | 19% | - | 14% | 19% | 5% | 9% | 5% |
| What is the minimum acceptable tools for follow up in patients with stage III-IV disease after curative treatment in an area with severe limited resources if computed tomography is not available? | Clinical examination only | CA 125 | Clinical examination + CA 125 | Pelvic and abdominal ultrasound and chest X-Ray + Physical exam | Pelvic and abdominal ultrasound and chest X-Ray + Physical exam + CA 125 | Pelvic and abdominal ultrasound and chest X-Ray + Physical exam + CA 125 + Vault cytology | - |
|  | 48% | - | 9% | 29% | 5% | 9% | - |
| What’s the minimum acceptable frequency of follow-up for recurrent endometrial cancer patients after response to salvage local therapy in an area with severe resources limitations? | Every 3 months in the first 2 years, after that, every six months until 5 years from the treatment | Every 6 months until 5 years from the treatment | Every 6 months in the first 2 years, adter that, annualy until 5 years from the treatment | None until the presence of symptoms | Abstain | Unqualified to answer | Annually until 5 years from the treatment |
|  | 84% | 5% | 11% | - | - | - | - |
| What is the minimum acceptable tools for follow up in patients with for recurrent endometrial cancer patients after response to salvage local therapy in an area with severe limited resources? | Clinical examination only | CA 125 | Clinical examination + CA 125 | Pelvic and abdominal ultrasound and chest X-Ray + Physical exam | Pelvic and abdominal ultrasound and chest X-Ray + Physical exam + CA 125 | Pelvic and abdominal computed tomography and chest X-Ray + CA 125 + Physical exam | Pelvic and abdominal computed tomography and chest X-Ray + CA 125 + Physical exam + Vault cytology |
|  | 15% | - | 10% | 5% | 20% | 25% | 5% |
| What is the minimum acceptable tools for follow up in patients with for recurrent endometrial cancer patients after response to salvage local therapy in an area with severe limited resources if computed tomography is not available? | Clinical examination only | CA 125 | Clinical examination + CA 125 | Pelvic and abdominal ultrasound and chest X-Ray + Physical exam | Pelvic and abdominal ultrasound and chest X-Ray + Physical exam + CA 125 | Pelvic and abdominal ultrasound and chest X-Ray + Physical exam + CA 125 + Vault cytology | - |
|  | 19% | - | 5% | 29% | 38% | 9% | - |

**Supplementary Table 4.** Questions related to surgical approach of endometrial cancer.

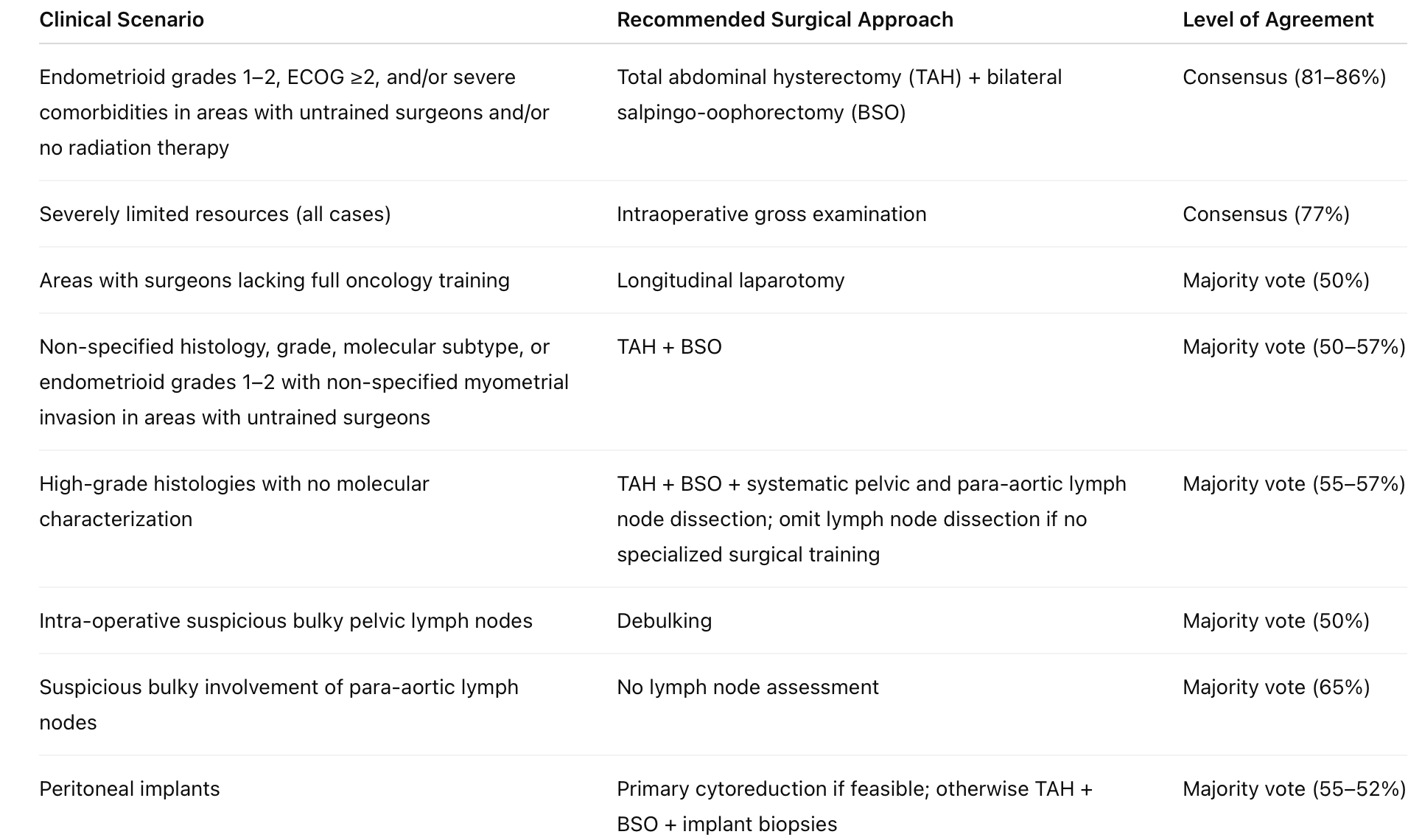

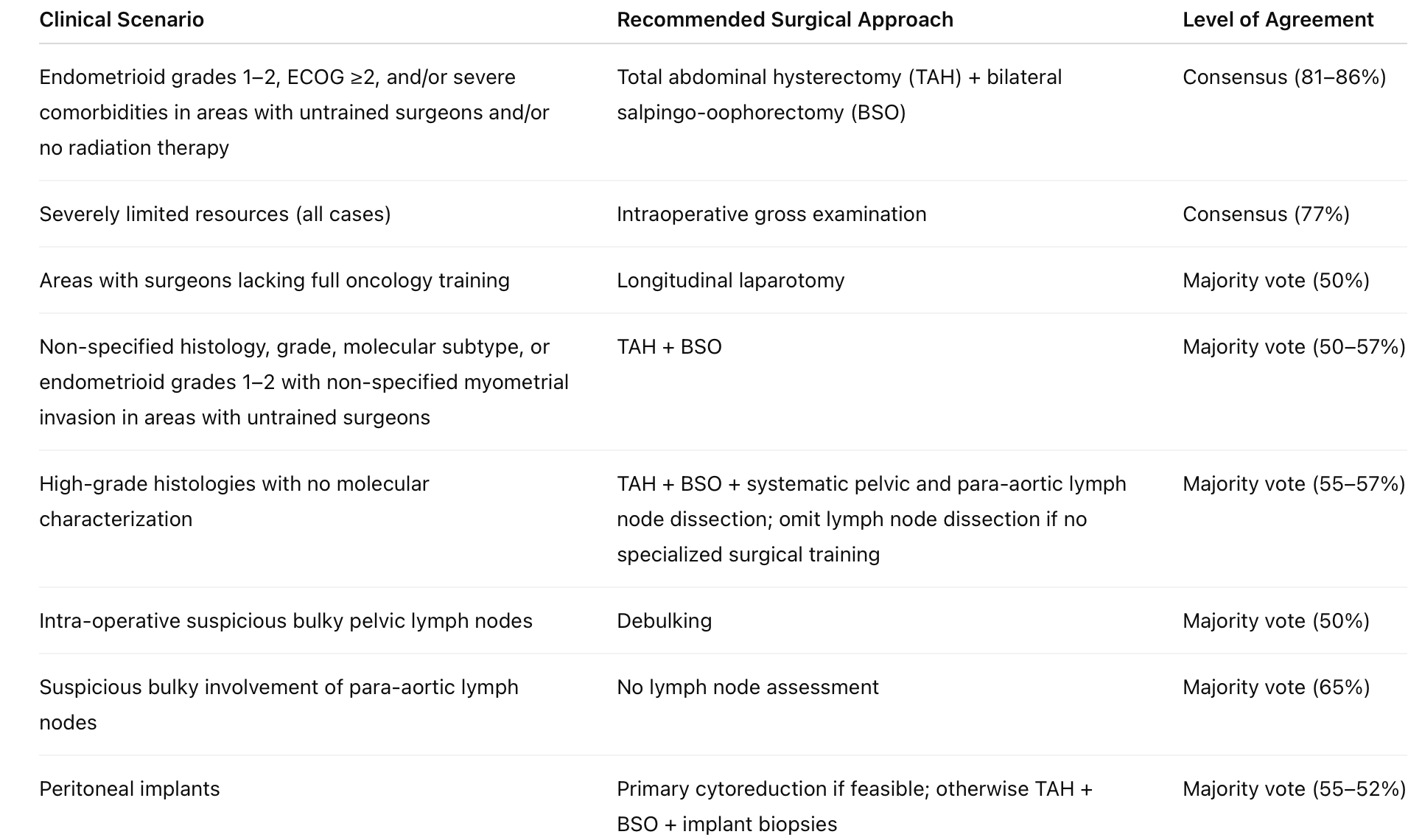


| Questions | **Responses** (%) | | | | | | |
| --- | --- | --- | --- | --- | --- | --- | --- |
| What type of incision is recommended to be done for surgical treatment of most endometrial cancer patients in an area of severe limited resources when surgeons do not have a full surgical oncology training? | Longitudinal laparotomy | All 3 are possible Pfannenstiel incision | Unqualified to answer | Pfannenstiel incision | Abstain | Vaginal Route | - |
|  | 50% | 35% | 10% | 5% | 0% | 0% | - |
| Is it useful to perform intraoperative gross examination by surgeons in an area of severe limited resources? | Yes, after surgeon’s training in surgical staging | Unqualified to answer | No, if it won’t change the surgical procedure | Abstain | - | - | - |
|  | 77% | 15% | 8% | 0% | - | - | - |
| What type of primary surgical approach should be performed for non-specified histologic type or grade or molecular subtype, regardless myometrial invasion in an area of severe limited resources? | Total Abdominal Hysterectomy (ysteHTAH) + Bilateral Salpingoophorectomy (BSO) + Pelvic lymph node sampling | TAH + BSO + Systematic pelvic lymph node dissection | TAH + BSO + Systematic pelvic and para-aortic lymph node dissection | TAH + BSO | TAH+ BSO + Pelvic and para-aortic lymph node sampling | Abstain | Unqualified to answer |
|  | 33% | 22% | 17% | 11% | 11% | 0% | 6% |
| What type of primary surgical approach should be performed for non-specified histologic type or grade or molecular subtype, regardless myometrial invasion in an area of severe limited resources when surgeons do not have a full surgical oncology training? | Total Abdominal Hysterectomy (ysteHTAH) + Bilateral Salpingoophorectomy (BSO) + Pelvic lymph node sampling | TAH + BSO + Systematic pelvic lymph node dissection | TAH + BSO + Systematic pelvic and para-aortic lymph node dissection | TAH + BSO | TAH+ BSO + Pelvic and para-aortic lymph node sampling | Abstain |  |
|  | 30% | 10% | 5% | 50% | 0% | 5% |  |
| What type of primary surgical approach should be performed for endometrioid grades 1 and 2 and non-specified myometrial invasion in an area of severe limited resources (no molecular characterization available)? | Total Abdominal Hysterectomy (ysteHTAH) + Bilateral Salpingoophorectomy (BSO) + Pelvic lymph node sampling | TAH + BSO + Systematic pelvic lymph node dissection | TAH + BSO + Systematic pelvic and para-aortic lymph node dissection | TAH + BSO | TAH+ BSO + Pelvic and para-aortic lymph node sampling | Abstain |  |
|  | 26% | 14% | 32% | 4% | 9% |  |  |
| What type of primary surgical approach should be performed for endometrioid grades 1 and 2 and non-specified myometrial invasion in an area of severe limited resources when surgeons do not have a full surgical oncology training (no molecular characterization available)? | Total Abdominal Hysterectomy (ysteHTAH) + Bilateral Salpingoophorectomy (BSO) + Pelvic lymph node sampling | TAH + BSO + Systematic pelvic lymph node dissection | TAH + BSO + Systematic pelvic and para-aortic lymph node dissection | TAH + BSO | TAH+ BSO + Pelvic and para-aortic lymph node sampling | Abstain |  |
|  | 29% | 9% | 5% | 57% | - | - |  |
| What type of primary surgical approach should be performed for high grade histologies in an area of severe limited resources (no molecular characterization available)? | Total Abdominal Hysterectomy (ysteHTAH) + Bilateral Salpingoophorectomy (BSO) + Pelvic lymph node sampling | TAH + BSO + Systematic pelvic lymph node dissection | TAH + BSO + Systematic pelvic and para-aortic lymph node dissection | TAH + BSO | TAH+ BSO + Pelvic and para-aortic lymph node sampling | Abstain |  |
|  | 5% | 25% | 55% | 5% | 5% | - |  |
| What type of primary surgical approach should be performed for high grade histologies in an area of severe limited resources when surgeons do not have a full surgical oncology training (no molecular characterization available)? | Total Abdominal Hysterectomy (ysteHTAH) + Bilateral Salpingoophorectomy (BSO) + Pelvic lymph node sampling | TAH + BSO + Systematic pelvic lymph node dissection | TAH + BSO + Systematic pelvic and para-aortic lymph node dissection | TAH + BSO | TAH+ BSO + Pelvic and para-aortic lymph node sampling | Abstain |  |
|  | 9% | 5% | 5% | 57% | 19% | - |  |
| In case of endometrioid grade 1 or 2 in a patient with ECOG 2 or more and/or severe comorbidities, what should be the best single approach in an area of severe limited resources? | Pelvic external beam radiotherapy | Total Vaginal Hysterectomy and Bilateral Salpingoophorectomy | Brachytherapy + Pelvic external beam radiotherapy | Oral Progestins | Brachytherapy | Supportive Care | TAH + BSO |
|  | - | 16% | 5% | 5% | - | - | 74% |
| In case of endometrioid grade 1 or 2 in a patient with ECOG 2 or more and/or severe comorbidities, what should be the best single approach in an area of severe limited resources when surgeons do not have a full surgical oncology training? | Pelvic external beam radiotherapy | Total Vaginal Hysterectomy and Bilateral Salpingoophorectomy | Brachytherapy + Pelvic external beam radiotherapy | Oral Progestins | Brachytherapy | Supportive Care | TAH + BSO |
|  | 5% | - | 9% | 5% | - | - | 81% |
| In case of endometrioid grade 1 or 2 in a patient with ECOG 2 or more and/or severe comorbidities, what should be the best single approach in an area of severe limited resources when radiation therapy is not available? | Pelvic external beam radiotherapy | Total Vaginal Hysterectomy and Bilateral Salpingoophorectomy | Brachytherapy + Pelvic external beam radiotherapy | Oral Progestins | Brachytherapy | Abstain | TAH + BSO |
|  |  | 9% |  | 5% |  | 5% | 82% |
| In case of endometrioid grade 1 or 2 in a patient with ECOG 2 or more and/or severe comorbidities, what should be the best single approach in an area of severe limited resources when surgeons do not have a full surgical oncology training and radiation therapy is not available? | Pelvic external beam radiotherapy | Total Vaginal Hysterectomy and Bilateral Salpingoophorectomy | Brachytherapy + Pelvic external beam radiotherapy | Oral Progestins | Brachytherapy | Abstain | TAH + BSO |
|  | - | 9% |  | 5% |  |  | 86% |
| 30.What surgical procedure should be done in case of intra-operative suspicious (bulky) pelvic lymph node in an area of severe limited resources when surgeons do not have a full surgical oncology training? |  | Do not resect the lymph node | Lymph node debulking | Lymph node debulking + para aortic lymph node sampling | Lymph node debulking + systematic pelvic lymph node dissection | Lymph node debulking + systematic pelvic and para-aortic lymph node dissection | Abstain/ Unqualified to answer |
|  |  | 18% | 50% | 5% | 5% | 9% | 13% |
| 31. What surgical procedure should be done in case of intra-operative suspicious (bulky) para-aortic lymph node in an area of severe limited resources when surgeons do not have a full surgical oncology training? |  | Do not resect the lymph node | Lymph node debulking | Lymph node debulking + para aortic lymph node sampling | Lymph node debulking + systematic pelvic lymph node dissection | Lymph node debulking + systematic pelvic and para-aortic lymph node dissection | Abstain/ Unqualified to answer |
|  |  | 65% | 22% | 5% | - | 8% |  |
| 32 What surgical approach in case of intra-operative peritoneal implants in an area of severe limited resources? |  | Only biopsy of peritoneal implants | TAH + BSO + Implant biopsies | Primary cytoreduction if surgically feasible | Abstain | Unqualified to answer |  |
|  |  | 9% | 27% | 55% | - | 9% |  |
| 33. What surgical approach in case of intra-operative peritoneal implants in an area of severe limited resources when surgeons do not have a full surgical oncology training? |  | Only biopsy of peritoneal implants | TAH + BSO + Implant biopsies | Primary cytoreduction if surgically feasible | Abstain | Unqualified to answer |  |
|  |  | 19% | 52% | 19% | - | 10% |  |

Legend: Answers to not all questions may total 100% due to rounding.

**Supplementary Table 5. Questions related to adjuvant treatment for endometrial cancer**

**
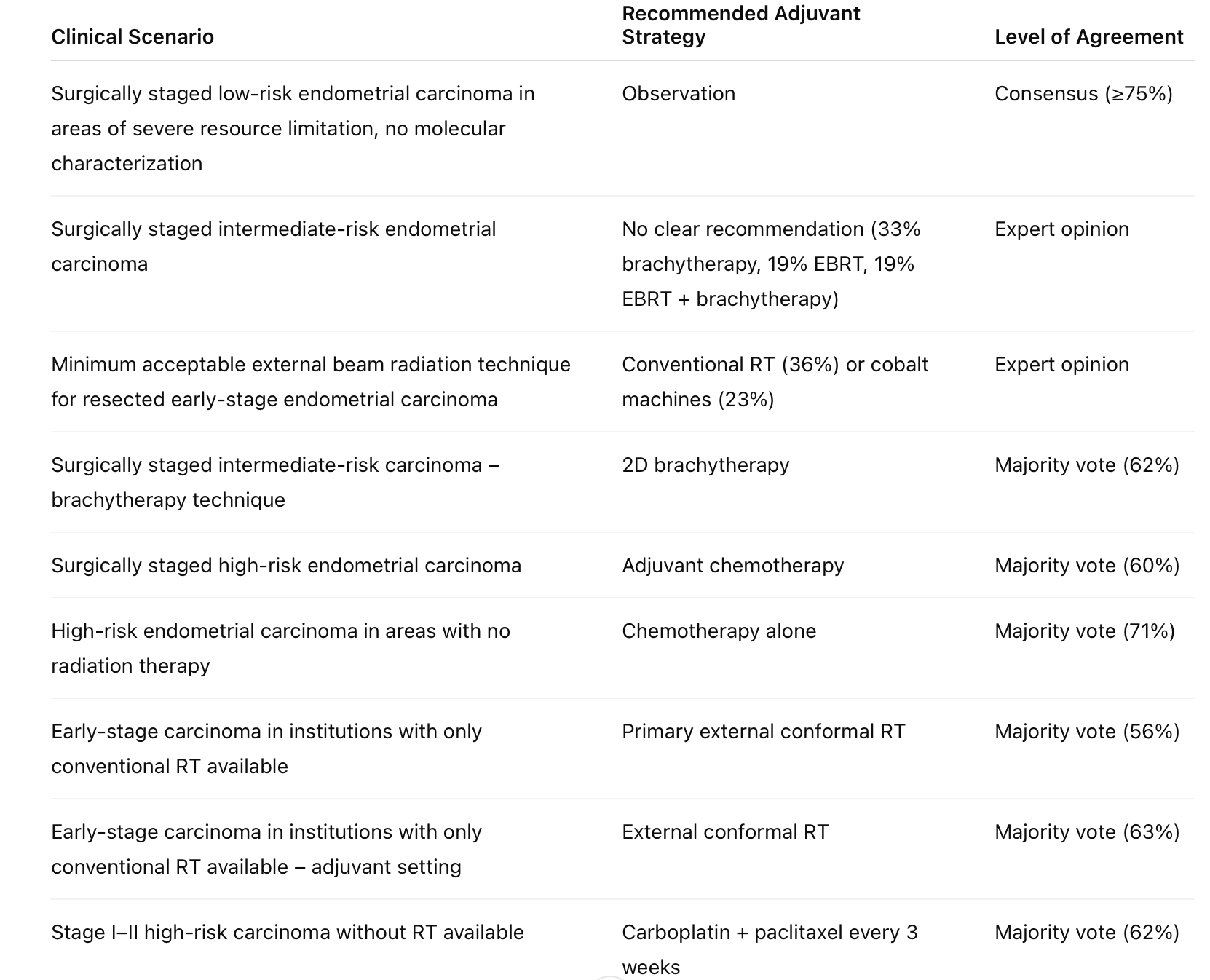
** **
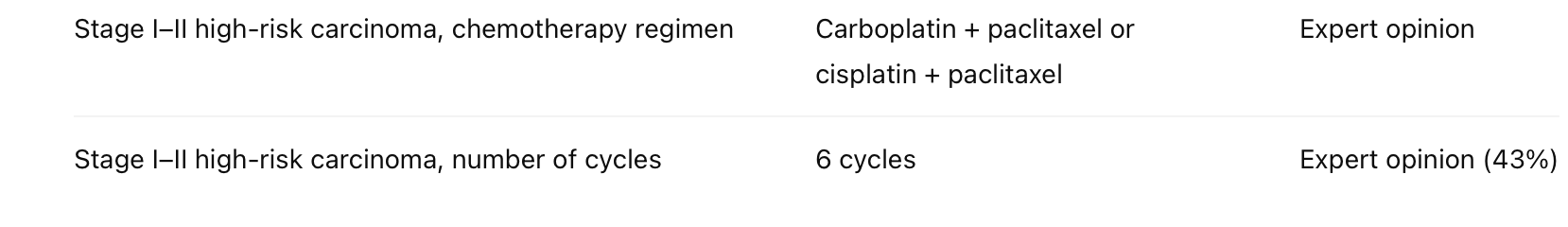
**

| Questions | **Responses (%)** | | | | | | |
| --- | --- | --- | --- | --- | --- | --- | --- |
| What is the minimum acceptable adjuvant strategy for surgically staged low risk endometrial carcinoma in areas of severe resources limitations (no molecular characterization available)? | | Observation | Radiation therapy (external) alone | Hormonal Therapy alone | Vaginal Brachytherapy | Observation | Abstain |
|  | | 90% | 10% | - | - | - | - |
| What is the minimum acceptable adjuvant strategy for surgically staged intermediate risk endometrial carcinoma in areas of severe resources limitations (no molecular characterization available)? | | Chemotherapy and Brachytherapy | Brachyterapy alone | Radiation therapy (external) alone | Radiation therapy (external) and brachyteraphy | Chemoterapy and radiation therapy (external) | Observation |
|  | | 5% | 33% | 19% | 19% | 10% | 14% |
| What is the minimum acceptable adjuvant strategy for surgically staged intermediate risk endometrial carcinoma in areas of severe resources limitations when radiation therapy is not available (no molecular characterization available)? | | Chemotherapy alone | Hormonal Therapy alone | Chemotherapy + Hormonal Therapy | Observation | Abstain |  |
|  | | 31% | 17% | 13% | 35% | 4% |  |
| What is the minimum acceptable adjuvant strategy for intermediate risk endometrial carcinoma, with no lymph node staging, in areas of severe resources limitations (no molecular characterization available)? | | Chemotherapy alone | Brachytherapy | Radiation therapy (external) alone | Radiation therapy (external) and brachytherapy | Chemotherapy and radiation therapy (external) | Abstain |
|  | | 11% | 11% | 42% | 26% | 5% | 5% |
| What is the minimum acceptable adjuvant external beam radiation technique to be offered for resected early stage endometrial carcinoma patients in areas of severe resources limitations? | | External beam Radiation Therapy with cobalt machines | Conventional radiation therapy | Conformal Radiation Therapy | Abstain | Unqualified to answer |  |
|  | | 23% | 36% | 5% | 9% | 27% |  |
| What is the minimum acceptable adjuvant brachytherapy technique to be offered for surgically staged intermediate risk endometrial carcinoma patients in areas of severe resources limitations? | | 2D | 3D | Abstain | Unqualified to answer |  |  |
|  | | 63% | 12% | 4% | 21% |  |  |
| If available, would you recommend adjuvant chemotherapy for surgically staged high risk endometrial carcinoma (no molecular characterization available)? | | Yes | No | Abstain | Unqualified to answer | Yes, only in cases with not adequate staging surgery |  |
|  | | 60% | 5% | 15% |  | 20% |  |
| What is the minimum acceptable adjuvant strategy for surgically staged high risk endometrial carcinoma in areas of severe resource limitations (no molecular characterization available)? | | Chemotherapy Alone | Radiation therapy (external) alone | Radiation therapy (external) and brachytherapy | Chemotherapy, Radiation therapy (external) and brachytherapy | Chemotherapy and radiation therapy (external) |  |
|  | | 17% | 22% | 11% | 28% | 22% |  |
| What is the minimum acceptable adjuvant strategy for surgically staged high risk endometrial carcinoma in areas of severe resources limitations when radiation therapy is not available (no molecular characterization available)? | | Chemotherapy alone | Hormonal Therapy alone | Chemotherapy plus hormonal therapy | Observation | Abstain |  |
|  | | 71% | 8% | 13% | 4% | 4% |  |
| In Institutions where there is only conventional radiotherapy technique, patients with early stages of endometrial cancer can be treated with primary external conformal radiotherapy? | | Yes | No | Abstain | Unqualified to answer |  |  |
|  | | 56% | 17% | 5% | 22% |  |  |
| In Institutions where there is only conventional radiotherapy technique, patients with early stages of endometrial cancer can be treated with adjuvant external conformal radiotherapy? | | Yes | No | Abstain | Unqualified to answer |  |  |
|  | | 63% | 4% | 8% | 25% |  |  |
| In Institutions where there is only cobalt machine, patients with early stages of endometrial cancer can be treated with primary external radiotherapy? | | Yes | No | Abstain | Unqualified to answer |  |  |
|  | | 41% | 41% | 4% | 14% |  |  |
| In Institutions where there is only cobalt machine, patients with early stages of endometrial cancer can be treated with adjuvant external radiotherapy? | | Yes | No | Abstain | Unqualified to answer |  |  |
|  | | 32% | 44% | 12% | 12% |  |  |
| What is the minimum acceptable chemotherapy regimen for adjuvant treatment in stage I-II high-risk endometrial cancer patients with no cisplatin contra-indication in areas of severe resources limitations? | | Cisplatin Alone or  Carboplatin Alone or  Cisplatin and doxorubicin or Abstain | Unqualified to answer | Cisplatin and paclitaxel every 3 weeks | Carboplatin and paclitaxel every 3 weeks |  |  |
|  | | 5% | 9% | 29% | 33% |  |  |
| What is the minimum acceptable chemotherapy regimen for adjuvant treatment in stage I-II high-risk endometrial cancer patients with no cisplatin contra-indication in areas of severe resources limitations and when radiation therapy is not available? | | Carboplatin and paclitaxel every 3 weeks | Cisplatin and paclitaxel every 3 weeks | Unqualified to answer | Abstain | None |  |
|  | | 62% | 19% | 9% | 5% | 5% |  |
| What is the minimum acceptable number of chemotherapy cycles for adjuvant treatment in stage I-II high-risk endometrial cancer patients in areas of severe resources limitations? | |  | Two | Three | Four | Six | Unqualified to answer |
|  | |  | 5% | 18% | 27% | 41% | 9% |
| What is the minimum acceptable number of chemotherapy cycles for adjuvant treatment in stage I-II high-risk endometrial cancer patients in areas of severe resources limitations when radiation therapy is not available? | |  | Three | Four | Five | Six | Unqualified to answer |
|  | |  | 19% | 19% | - | 43% | 19% |
| What is the minimum acceptable salvage systemic regimen for platinum resistant/refractory endometrial cancer patients NOT exposed to taxanes in areas of severe resources limitations? | | Gencitabine | Doxorrubicin | Paclitaxel weekly | Hormone therapy | Topotecan or Etoposide | Abstain and Unqualified to answer |
|  | | 11% | 5% | 42% | 16% | - | 26% |

Legend: Answers to not all questions may total 100% due to rounding.

**Supplementary Table 6. Questions related to first-line treatment of metastatic or locally advanced endometrial cancer**

**
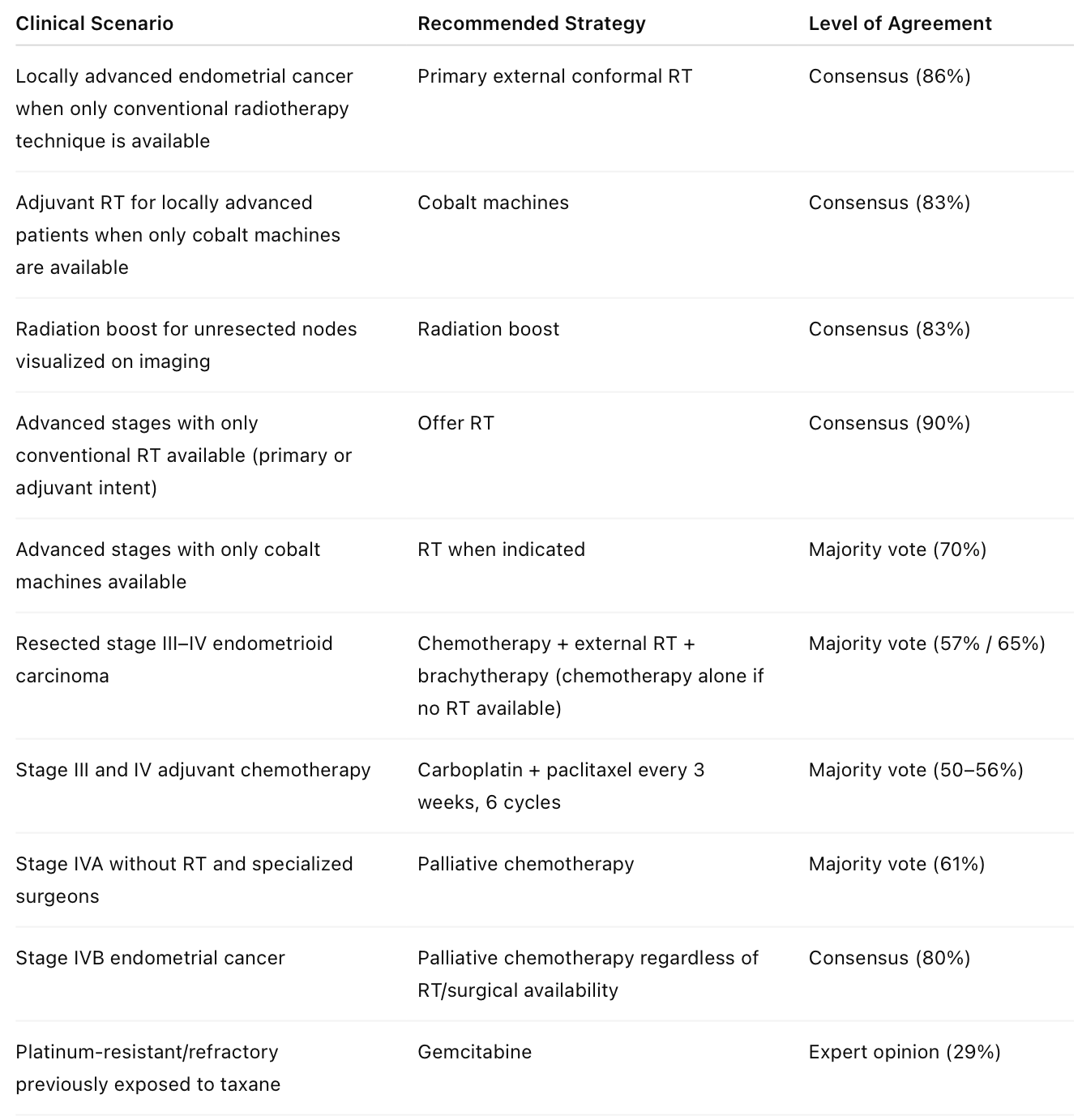
** **
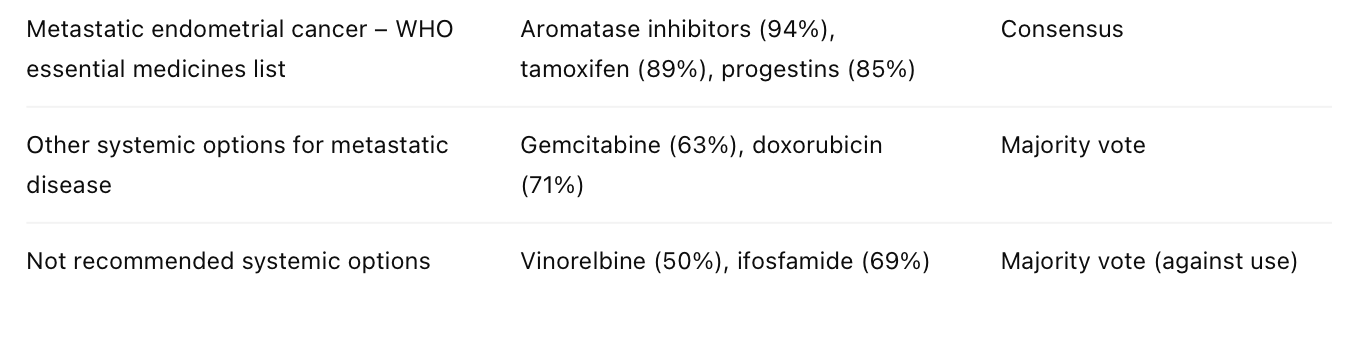
**

| Questions | | **Responses** (%) | | | | | | |
| --- | --- | --- | --- | --- | --- | --- | --- | --- |
| What should be the best treatment approach for stage IVa endometrial cancer (bladder or rectum invasion) in an area of severe limited resources? |  | Brachytherapy + Pelvic external beam radiotherapy or Pelvic radiotherapy followed by surgery | Primary pelvic exenteration if technically feasible or Pelvic radiotherapy followed by surgery and chemotherapy sequentially | Neoadjuvant chemotherapy followed by surgery | Pelvic radiotherapy | Primary treatment with chemoradiotherapy | Abstain |  |
|  |  | 5% | 14% | 29% | 10% | 14% | 9% |  |
| What should be the best treatment approach for stage IVa endometrial cancer (bladder or rectum invasion) in an area of severe limited resources when radiation therapy is available? |  | Pelvic radiotherapy followed by surgery or Pelvic radiotherapy | Chemotherapy | Neoadjuvant chemotherapy followed by surgery or Brachytherapy + Pelvic external beam radiotherapy | Abstain/ Unqualified to answer | Primary treatment with chemoradiotherapy or Pelvic radiotherapy followed by surgery and chemotherapy sequentially | Brachytherapy + Pelvic external beam radiotherapy |  |
|  |  | 5% | 9% | 14% | 12% | 23% | 14% |  |
| What should be the best treatment approach for stage IVa endometrial cancer (bladder or rectum invasion) in an area of severe limited resources when radiation therapy are is available? |  | Primary pelvic exenteration if techinically feasible | Neoadjuvant chemotherapy followed by surgery | Palliative chemotherapy | Palliative hormonal therapy | Abstain |  |  |
|  |  | 26% | 44% | 22% | 4% | 4% |  |  |
| What should be the best treatment approach for stage IVa endometrial cancer (bladder or rectum invasion) in an area of severe limited resources when surgeons do not have a full surgical oncology training and when radiation therapy are not available? |  | Primary pelvic exenteration if techinically feasible | Neoadjuvant chemotherapy followed by surgery | Palliative chemotherapy | Palliative hormonal therapy | Abstain |  |  |
|  |  | 13% | 18% | 61% | 4% | 4% |  |  |
| What is the minimum acceptable adjuvant strategy for resected stage III or IV uterine endometrioid carcinoma in areas of severe resources limitations (no molecular characterization available)? |  | Chemotherapy alone | Radiation therapy (external) and brachytherapy | Chemotherapy and Radiation therapy(external) and brachytherapy | Chemotherapy and radiation therapy (external) | Brachytherapy alone | Chemotherapy and brachytherapy |  |
|  |  | 57% | 5% | 9% | 24% | - | - |  |
| What is the minimum acceptable adjuvant strategy for resected stage III or IV uterine endometrioid carcinoma in areas of severe resources limitations when radiation therapy is not available (no molecular characterization available)? |  | Chemotherapy alone | Hormonal therapy alone | Chemotherapy plus hormonal therapy | Observation | Abstain | Unqualified to answer |  |
|  |  | 65% | - | 26% | - | 5% | 4% |  |
| What is the minimum acceptable adjuvant external beam radiation technique for resected stage III or IV uterine endometrioid carcinoma in areas of severe resources limitations? |  | External beam radiation therapy with cobalt machines | Conventional radiation therapy | Conformal radiation therapy | Abstain | Unqualified to answer |  |  |
|  |  | 17% | 42% | 8% | 8% | 25% |  |  |
| In Institutions where there is only conventional radiotherapy technique, patients with locally advanced endometrial cancer can be treated with primary external conformal radiotherapy? |  | Yes | No | Unqualified to answer | Abstain |  |  |  |
|  |  | 54% | 25% | 21% | - |  |  |  |
| In Institutions where there is only conventional radiotherapy technique, patients with locally advanced endometrial cancer can be treated with adjuvant external conformal radiotherapy? |  | Yes | No | Unqualified to answer | Abstain |  |  |  |
|  |  | 86% | - | 14% | - |  |  |  |
| In Institutions where there is only cobalt machine, patients with locally advanced endometrial cancer can be treated with primary external radiotherapy? |  | Yes | No | Unqualified to answer | Abstain |  |  |  |
|  |  | 58% | 17% | 25% | - |  |  |  |
| In Institutions where there is only cobalt machine, patients with locally advanced endometrial cancer can be treated with adjuvant external radiotherapy? |  | Yes | No | Unqualified to answer | Abstain |  |  |  |
|  |  | 83% | 4% | 13% | - |  |  |  |
| For patients with unresected nodes that are visualized on imaging, a radiation dose “boost” should be delivered as means to achieve better local control, in areas of severe resources limitations? |  | Yes | No | Unqualified to answer | Abstain |  |  |  |
|  |  | 83% | 4% | 13% | - |  |  |  |
| What is the minimum technique is required to deliver a radiation dose “boost” to suspicious nodes in areas of severe resources limitations? |  | External beam Radiation Therapy with Cobalt machines | Conventional Radiation Therapy | Conformal Radiation Therapy | Abstain | Unqualified to answer |  |  |
|  |  | 9% | 26% | 22% | 4% | 39% | - |  |
| In Institutions where there is only conventional radiotherapy technique, patients with advanced stages of endometrial cancer can be treated with primary or adjuvant external radiotherapy? |  | Yes | No | Unqualified to answer | Abstain |  |  |  |
|  |  | 90% | - | 10% | - |  |  |  |
| In Institutions where there is only cobalt machine, patients with advanced stages of endometrial cancer can be treated with primary or adjuvant external radiotherapy? |  | Yes | No | Unqualified to answer | Abstain |  |  |  |
|  |  | 70% | 10% | 15% | 5% |  |  |  |
| What is the minimum acceptable chemotherapy regimen for adjuvant treatment in stage III endometrial cancer patients with no cisplatin contra-indication in areas of severe resources limitations (no molecular characterization available)? |  | Cisplatin alone | Carboplatin alone | Cisplatin and paclitaxel every 3 weeks | Carboplatin and paclitaxel every 3 weeks | Carboplatin and paclitaxel weekly | Abstain and Unqualified to answer |  |
|  |  | - | 5% | 25% | 50% | - | 20% |  |
| What is the minimum acceptable number of chemotherapy cycles for adjuvant treatment in stage III endometrial cancer patients in areas of severe resources limitations? |  | One or Two | Three | Four | Five | Six | Abstain/ Unqualified to answer |  |
|  |  | - | 21% | 21% | - | 58% | 19% |  |
| What should be the best treatment approach for stage IVb endometrial cancer (visceral disease) in an area of severe limited resources? |  | Hormone therapy if low grade | Pelvic Radiotherapy | TAH + BSO + Chemotherapy | Pelvic Radiotherapy + Chemotherapy | Palliarive chemotherapy | Supportive care without oncological treatment |  |
|  |  | 5% | 6% | 11% | 11% | 61% | 6% |  |
| What should be the best treatment approach for stage IVb endometrial cancer (visceral disease) in an area of severe limited resources when surgeons do not have a full surgical oncology training? |  | Pelvic radiotherapy | TAH + BSO + Chemotherapy | Palliative chemotherapy | Supportive csare without oncological treatment | TAH + BSO |  |  |
|  |  | 10% | 10% | 70% | 10% | - |  |  |
| What should be the best treatment approach for stage IVb endometrial cancer (visceral disease) in an area of severe limited resources when radiation therapy are not available? |  | TAH + BSO + Chemotherapy | Pelvic radiotherapy + Chemotherapy | Palliative chemotherapy | Supportive care without oncological treatment |  |  |  |
|  |  | 14% | 9% | 68% | 9% |  |  |  |
| What should be the best treatment approach for stage IVb endometrial cancer (visceral disease) in an area of severe limited resources when surgeons do not have a full surgical oncology training and when radiation therapy are not available? |  | TAH + BSO | TAH + BSO + Chemotherapy | Pelvic radiotherapy + Chemotherapy | Palliative chemotherapy | Supportive care without oncological treatment |  |  |
|  |  | - | 5% | 10% | 80% | 5% |  |  |
| What is the minimum acceptable chemotherapy regimen for treatment in stage IV endometrial cancer patients with no cisplatin contra-indication in areas of severe resources limitations? |  | Carboplatin and paclitaxel every 3 weeks | Cisplatin and paclitaxel every 3 weeks | Carboplatin alone | Cisplatin alone | Carboplatin weekly and paclitaxel weekly | Unqualified to answer |  |
|  |  | 45% | 14% | 14% | - | - | 27% |  |
| What is the minimum acceptable chemotherapy regimen for treatment in stage IV endometrial cancer patients with important comorbidities and/or cisplatin contra- indication in areas of severe resources limitations? |  | Carboplatin alone | Carboplatin weekly and paclitaxel weekly | No platinum regimen | Abstain | Unqualified to answer | Carboplatin and paclitaxel every 3 weeks |  |
|  |  | 14% | 10% | 14% | 9% | 24% | - |  |
| What is the minimum acceptable number of chemotherapy cycles for treatment in stage IV endometrial cancer patients in areas of severe resources limitations? |  | One or Two or Three | Abstain and Unqualified to answer | Four | Five | Six | Eight |  |
|  |  | - | 14% | 14% | - | 67% | 5% |  |
| What is your first-line treatment for patients with advanced endometrial cancer without access to taxanes or where taxane-related costs are prohibitive? |  | Platinum and 5-fluorouracil | Platinum and cyclophosphamide | Platinum and doxorubicin (not liposomal) | Cisplatin | Carboplatin | Abstain and Unqualified to answer |  |
|  |  | 5% | 9% | 33% | 5% | 24% | 24% |  |
| Each of the following drugs is on the WHO essential medicines list. You are able to purchase them at an affordable price from generic manufacturer. Which would you consider as appropriate treatment options for women with metastatic endometrial cancer in the setting of limited healthcare resources? – Ifosfamide |  | Yes | No | Abstain | Unqualified to answer |  |  |  |
|  |  | - | 69% | 6% | 25% |  |  |  |
| Each of the following drugs is on the WHO essential medicines list. You are able to purchase them at an affordable price from generic manufacturer. Which would you consider as appropriate treatment options for women with metastatic endometrial cancer in the setting of limited healthcare resources? – Topotecan |  | Yes | No | Abstain | Unqualified to answer |  |  |  |
|  |  | 40% | 25% | - | 35% |  |  |  |
| Each of the following drugs is on the WHO essential medicines list. You are able to purchase them at an affordable price from generic manufacturer. Which would you consider as appropriate treatment options for women with metastatic endometrial cancer in the setting of limited healthcare resources? – Doxorubicin |  | Yes | No | Abstain | Unqualified to answer |  |  |  |
|  |  | 71% | - | - | 29% |  |  |  |
| Each of the following drugs is on the WHO essential medicines list. You are able to purchase them at an affordable price from generic manufacturer. Which would you consider as appropriate treatment options for women with metastatic endometrial cancer in the setting of limited healthcare resources? – Gemcitabine |  | Yes | No | Abstain | Unqualified to answer |  |  |  |
|  |  | 63% | 5% | 5% | 27% |  |  |  |
| Each of the following drugs is on the WHO essential medicines list. You are able to purchase them at an affordable price from generic manufacturer. Which would you consider as appropriate treatment options for women with metastatic endometrial cancer in the setting of limited healthcare resources? – Cyclophosphamide |  | Yes | No | Abstain | Unqualified to answer |  |  |  |
|  |  | 45% | 30% | - | 25% |  |  |  |
| Each of the following drugs is on the WHO essential medicines list. You are able to purchase them at an affordable price from generic manufacturer. Which would you consider as appropriate treatment options for women with metastatic endometrial cancer in the setting of limited healthcare resources? – 5-FU |  | Yes | No | Abstain | Unqualified to answer |  |  |  |
|  |  | 32% | 37% | - | 31% |  |  |  |
| Each of the following drugs is on the WHO essential medicines list. You are able to purchase them at an affordable price from generic manufacturer. Which would you consider as appropriate treatment options for women with metastatic endometrial cancer in the setting of limited healthcare resources? – Vinorelbine |  | Yes | No | Abstain | Unqualified to answer |  |  |  |
|  |  | 17% | 50% | - | 33% |  |  |  |
| Each of the following drugs is on the WHO essential medicines list. You are able to purchase them at an affordable price from generic manufacturer. Which would you consider as appropriate treatment options for women with metastatic endometrial cancer in the setting of limited healthcare resources? – Etoposide |  | Yes | No | Abstain | Unqualified to answer |  |  |  |
|  |  | 22% | 39% | 6% | 33% |  |  |  |
| Each of the following drugs is on the WHO essential medicines list. You are able to purchase them at an affordable price from generic manufacturer. Which would you consider as appropriate treatment options for women with metastatic endometrial cancer in the setting of limited healthcare resources? – Progestins |  | Yes | No | Abstain | Unqualified to answer |  |  |  |
|  |  | 85% | 5% | - | 10% |  |  |  |
| Each of the following drugs is on the WHO essential medicines list. You are able to purchase them at an affordable price from generic manufacturer. Which would you consider as appropriate treatment options for women with metastatic endometrial cancer in the setting of limited healthcare resources? – Tamoxifen |  | Yes | No | Abstain | Unqualified to answer |  |  |  |
|  |  | 89% | 6% | - | 5% |  |  |  |
| Each of the following drugs is on the WHO essential medicines list. You are able to purchase them at an affordable price from generic manufacturer. Which would you consider as appropriate treatment options for women with metastatic endometrial cancer in the setting of limited healthcare resources? – Aromatase inhibitor |  | Yes | No | Abstain | Unqualified to answer |  |  |  |
|  |  | 94% | - | - | 6% |  |  |  |

Answers to not all questions may total 100% due to rounding.

**Supplementary Table 7. Questions related to recurrent endometrial cancer**

**
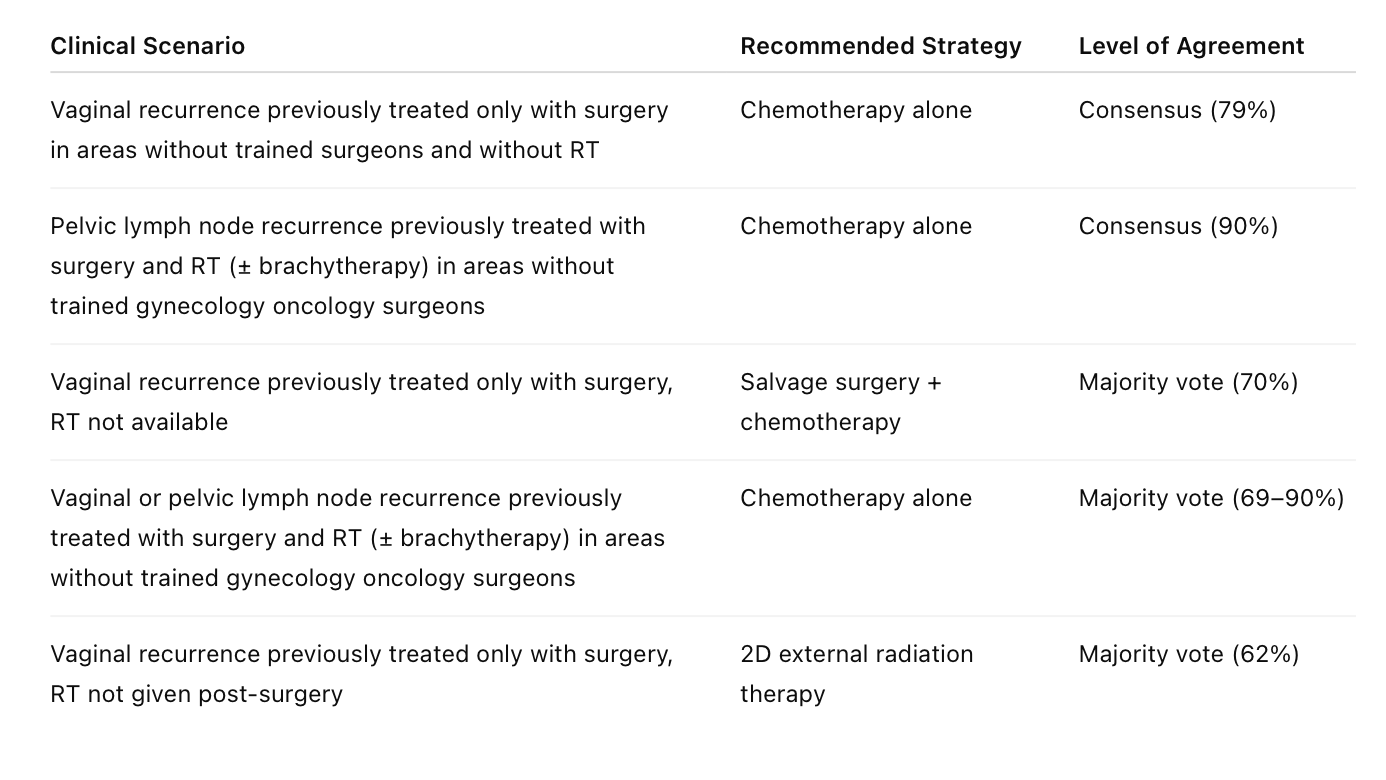
**

| Questions | | **Responses** (%) | | | | | | |
| --- | --- | --- | --- | --- | --- | --- | --- | --- |
| What is the minimum acceptable salvage treatment regimen for recurrent platinum sensitive endometrial cancer patients in areas of severe resources limitations? |  | Cisplatin alone | Carboplatin alone | Cisplatin and paclitaxel every 3 weeks or cisplatin and doxorubicin (not liposomal) | Carboplatin and paclitaxel every 3 weeks | Non-platinum regimen or Hormone therapy if low grade disease | Abstain and Unqualified to answer |  |
|  |  | - | 5% | 14% | 33% | 5% | 24% |  |
| What is the minimum acceptable salvage treatment regimen for recurrent platinum sensitive endometrial cancer patients with severe comorbidities in areas of severe resources limitations? |  | Carboplatin alone | Cisplatin and paclitaxel every 3 weeks | Non platinum regimen | Hormone therapy, if low grade disease | Abstain | Unqualified to answer |  |
|  |  | 13% | 17% | 9% | 43% | 9% | 9% |  |
| What is the minimum acceptable number of chemotherapy cycles for recurrent platinum sensitive endometrial cancer patients in areas of severe resources limitations? |  | One or Two | Three | Four | Five | Six | Abstain and Unqualified |  |
|  |  | - | 9% | 18% | - | 54% | 19% |  |
| What is the minimum acceptable salvage systemic regimen for recurrent platinum resistant/refractory endometrial cancer patients exposed to taxanes in areas of severe resources limitations? |  | Gencitabine | Doxorrubicin | Paclitaxel weekly | Hormone therapy | Topotecan or Etoposide | Abstain and Unqualified to answer |  |
|  |  | 29% | 19% | - | 19% | - | 33% |  |
| What is the minimum acceptable salvage systemic regimen for recurrent platinum resistant/refractory endometrial cancer patients with severe comorbidities exposed to taxanes in areas of severe resources limitations? |  | Gencitabine | Doxorrubicin | Paclitaxel weekly | Hormone therapy | Topotecan or Etoposide | Abstain and Unqualified to answer |  |
|  |  | 14% | 14% | - | 38% | - | 34% |  |
| What is your hormone therapy of choice for the treatment of advanced or recurrent endometrial cancer patients in areas of severe resources limitations? |  | Aromatase Inhibitor | Progestins | Tamoxifen alternated with progestins | Tamoxifen | Abstain and Unqualified to answer |  |  |
|  |  | 16% | 42% | 16% | - | 26% |  |  |
| For women with advanced, recurrent, and platinum resistant/refractory endometrial cancer with no clinical trial available, when do you recommend best supportive care in an area with limited resources? |  | After first-line treatment | After second-line treatment | After third-line treatment or more | Performance status > 2, unrelated to line of treatment | Abstain and Unqualified to answer |  |  |
|  |  | 5% | 24% | 5% | 57% | 9% |  |  |
| What is the minimum acceptable strategy for vaginal recurrence from endometrial cancer treated only with surgery in areas of severe resources limitations? |  | Chemotherapy alone or Hormonal therapy alone | Salvage surgery | Salvage surgery and chemotherapy | Brachytherapy | Radiation therapy (external) or radiation therapy and chemotherapy | Abstain and unqualified to answer |  |
|  |  | - | 11% | 27% | 21% | 5% | 10% |  |
| What is the minimum acceptable strategy for vaginal recurrence from endometrial cancer treated only with surgery in areas of severe resources limitations when radiation therapy is not available? |  | Chemotherapy alone or Hormonal therapy alone | Salvage surgery | Salvage surgery and chemotherapy | Abstain |  |  |  |
|  |  | - | 25% | 70% | 5% |  |  |  |
| What is the minimum acceptable strategy for vaginal recurrence from endometrial cancer treated only with surgery in areas of severe resources limitations when radiation therapy is not available and when surgeons do not have a full training in gynecology oncology? |  | Chemotherapy alone | Salvage surgery | Salvage surgery and chemotherapy | Hormonal therapy alone | Abstain or Unqualified to answer |  |  |
|  |  | 79% | - | 10% | 11% | - |  |  |
| What is the minimum acceptable radical RT strategy for patients with vaginal recurrence of uterine endometrioid carcinoma, who did not received radiation after surgery, in areas of severe resources limitations? |  | Cobalt therapy alone | 2D radiation therapy alone (external) | 3D Radiation Therapy Alone (external) | Abstain | Unqualified to answer |  |  |
|  |  | 10% | 62% | 9% | - | 19% |  |  |
| What is the minimum acceptable strategy for vaginal recurrence from endometrial cancer treated previously with surgery and brachytherapy in areas of severe resources limitations? |  | Chemotherapy alone | Horomonal therapy alone | Salvage surgery followed by chemotherapy | Radiation therapy (external) | Chemotherapy and radiation therapy (external) | Abstain and unqualified to answer |  |
|  |  | 30% | - | 40% | 20% | 5% | - |  |
| What is the minimum acceptable strategy for vaginal recurrence from endometrial cancer treated previously with surgery and radiation therapy (external) +/- brachytherapy in areas of severe resources limitations? |  | Chemotherapy alone | Hormonal therapy alone | Salvage surgery and chemotherapy | Re-radiation therapy (external) | Chemotherapy and re-radiation therapy (external) | Abstain or Unqualified |  |
|  |  | 20% | 15% | 45% | - | - | - |  |
| What is the minimum acceptable strategy for vaginal recurrence from endometrial cancer treated previously with surgery and radiation therapy (external) and/or brachytherapy in areas of severe resources limitations when surgeons do not have a full training in gynecology oncology? |  | Chemotherapy alone | Hormonal therapy alone | Salvage surgery and chemotherapy | Re-radiation therapy (external) | Chemotherapy and re-radiation therapy (external) | Salvage surgery |  |
|  |  | 69% | 16% | 5% | - | 5% | 5% |  |
| What is the minimum acceptable strategy for pelvic lymph node recurrent endometrial cancer treated previously with surgery and radiation therapy (external) +/- brachytherapy in areas of severe resources limitations? |  | Chemotherapy alone | Hormonal therapy alone | Salvage surgery and chemotherapy | Re-radiation therapy (external) | Chemotherapy and re-radiation therapy (external) | Salvage surgery |  |
|  |  | 53% | - | 37% | - | 10% | - |  |
| What is the minimum acceptable strategy for pelvic lymph node recurrence from endometrial cancer treated previously with surgery and radiation therapy (external) +/- brachytherapy in areas of severe resources limitations when surgeons do not have a full training in gynecology oncology? |  | Chemotherapy alone | Hormonal therapy alone | Salvage surgery and chemotherapy | Re-radiation therapy (external) | Chemotherapy and re-radiation therapy (external) | Salvage surgery |  |
|  |  | 90% | - | 10% | - | - | - |  |

Answers to not all questions may total 100% due to rounding.
